# Genetic structure of two sympatric gudgeon fishes (*Xenophysogobio boulengeri* and *X. nudicorpa*) in the upper reaches of Yangtze River Basin

**DOI:** 10.7717/peerj.7393

**Published:** 2019-08-06

**Authors:** Weiwei Dong, Dengqiang Wang, Huiwu Tian, Yan Pu, Lixiong Yu, Xinbin Duan, Shaoping Liu, Daqing Chen

**Affiliations:** 1 Key Laboratory of Freshwater Fish Reproduction and Development (Ministry of Education), Key Laboratory of Aquatic Science of Chongqing, School of Life Sciences, Southwest University, Chongqing, China; 2 Yangtze River Fisheries Research Institute, Chinese Academy of Fishery Science, Wuhan, China

**Keywords:** Genetic structure, Sympatric fish, Geographic genetic subdivision, *Xenophysogobio*, Genetic diversity

## Abstract

**Background:**

*Xenophysogobio boulengeri* and *X. nudicorpa* are the only two species within the genus *Xenophysogobio* (Cyprinidae, Cypriniformes), and both are endemic to the upper reaches of the Yangtze River. In recent years, due to human activities, the natural resources available to both species have declined sharply. Sympatric species with overlapping niches inevitably compete for their habitats, and genetic structure and diversity can reflect population history and their potential for adaptation to changing environments, which is useful for management decisions.

**Methods:**

In the present study, microsatellite DNA and mitochondrial DNA (mtDNA) markers were used to investigate the patterns of population genetic structure for *X. boulengeri* and *X. nudicorpa*. Microsatellite DNA data, jointly with traditional summary statistics including *F*_ST_ and *F*_is_, were used to assess the population genetic structure by structure analysis. The mtDNA sequences were then used to examine these patterns through time to detect demographic history.

**Results:**

*Xenophysogobio boulengeri* and *X. nudicorpa* exhibited high levels of genetic diversity in Yangtze River populations, except for two populations of *X. nudicorpa* in the Jinsha River, which were low in mtDNA diversity. *X. boulengeri* showed genetic homogeneity among populations, whereas *X. nudicorpa* appeared to have significant geographic genetic divergence. Both species experienced a late-Pleistocene sudden population expansion in Yangtze River populations, but not in the Jinsha River populations of *X. nudicorpa*.

**Discussion:**

The genetic homogeneity of *X. boulengeri* populations might result from similar population expansion events and environment features. The geographic genetic subdivision for *X. nudicorpa* between the Jinsha and Yangtze Rivers might be caused by the geographic isolation in the middle Pliocene, as well as climate and environmental heterogeneity.

## Introduction

**Figure 1:**
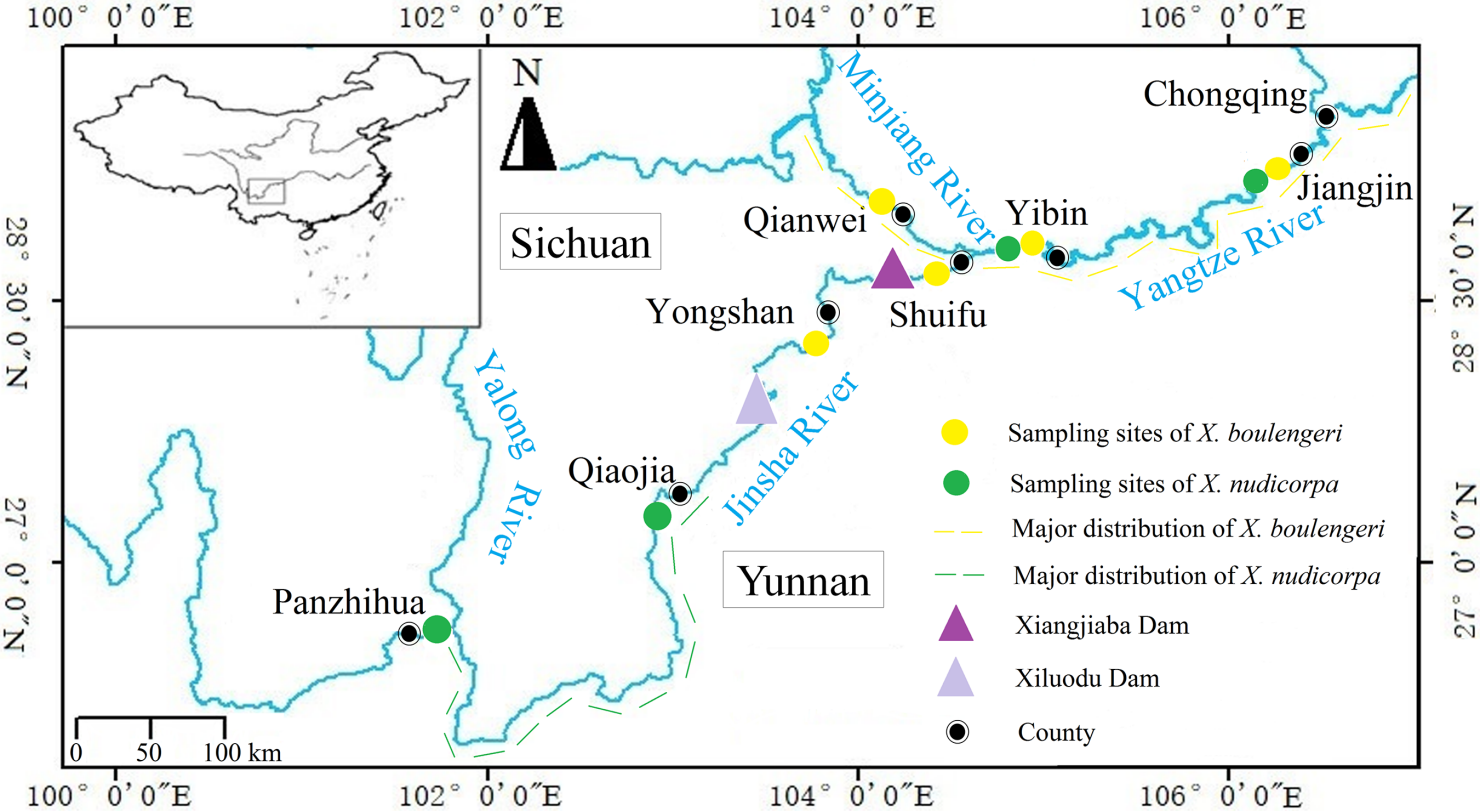
Sampling locations for *X. boulengeri* and *X. nudicorpa* within the upper reaches of Yangtze River Basin. This figure shows collection sites and major distribution of *X. boulengeri* and *X. nudicorpa*.

The Yangtze River is the longest river in China and the third longest river in the world. It originates from the Qinghai-Tibetan Plateau and follows a sinuous easterly route before emptying into the East China Sea at Shanghai city. The section above Yichang city in the Huhei province (the Three Gorges Dam site) is generally regarded as the upper reaches and is characterized by mountains, raging torrents and a high altitude compared with the middle and lower reaches. The section above Yibin (YB) city in the Sichuan Province, known as the Jinsha River (Fig. 1), is the origin of the Yangtze River and is characterized by narrow, swift currents. In addition to changing altitudes and physical geographical features, water temperatures and dissolved oxygen levels change considerably along the different stream segments, which can affect the metabolism, reproduction and community distributions of fish (Shi et al., 2018). The upper reaches of the Yangtze River represent a biodiversity hotspot, with more than 200 fish species present, 70 of which are endemic (Wei, 2012). These fish have adapted to the local environment and are sensitive to environmental changes (Luo et al., 2014). Compared to the upper reaches of the Yangtze River, which is influenced by a typical subtropical climate (Table 1), the Jinsha River runs through a higher altitude landscape and is influenced by a temperate, three-dimensional climate. There are many species of fish in the upper reaches of the Yangtze River, while the level of fish species diversity is low in the Jinsha River.

**Table 1:** Sampling sites for *X. boulengeri* and *X. nudicorpa*, coordinates, altitude and climate.

Sample site	ID	Drainage	Coordinates	Altitude (m)	Climate
Jiangjin, Chongqing	JJ	Yangtze River	106°16′48″E, 29°16′48″N	172	Subtropical monsoon humid climate
Yibin, Sichuan	YB	Yangtze River	104°41′24″E, 28°46′12″N	283	Subtropical monsoon humid climate
Qianwei, Sichuan	QW	Minjiang River	103°55′48″E, 29°13′48″N	321	Subtropical monsoon climate
Shuifu, Yunnan	SF	Jinsha River	104°25′12″E, 28°37′48″N	268	Subtropical humid climate
Yongshan, Yunnan	YS	Jinsha River	103°40′12″E, 28°14′24″N	434	Temperate monsoon climate
Qiaojia, Yunnan	QJ	Jinsha River	102°58′48″E, 26°48′N	679	Temperate climate
Panzhihua, Sichuan	PZH	Jinsha River	101°45′E, 26°34′12″N	1,050	A three-dimensional climate based on the south subtropical zone

The subfamily Gobiobotinae (Cyprinidae, Cypriniformes) is a group of small freshwater fish, distributed across East Asia, including Korea and China (Chen, 1998). There are only two genera, *Gobiobotia* and *Xenophysogobio*, in this subfamily with about 17 species of which *Xenophysogobio* represent the primitive species (He, 1991; Wang, He & Chen, 2002). *Xenophysogobio boulengeri* and *X. nudicorpa* are the only two species in the *Xenophysogobio* genus, and both are endemic to the upper reaches of the Yangtze River (Ding, 1994). They share similar geographical distributions, but with some differences in their centers of abundance. For *X. boulengeri*, the centers of abundance are in upper streams of the Yangtze and Minjiang rivers (Gao, 2016; Xiong et al., 2015; Lv et al., 2018), while *X. nudicorpa* is dominant in the Qiaojia (QJ)-Panzhihua (PZH) section (Gao et al., 2013) (Fig. 1). They represent two closely related species and both exhibit many similar features, such as producing drifting eggs, benthic living and feeding on invertebrates (Ding, 1994), which indicates that they occupy similar ecological niches. Competitive pressures exist among ecologically similar species, which can change the population structure of both species (Förschler et al., 2009). For instance, competition can cause demographic changes and one of the two species may experience a demographic drop. Therefore, we want to test whether the population genetic structures of *X. boulengeri* and *X. nudicorpa* would develop similarly or differently in the same environment. At present, the abundance of *X. nudicorpa* is lower than *X. boulengeri*, possibly suggesting that *X. nudicorpa* experiences a competitive disadvantage. Our study further sought to investigate whether differences in genetic diversity and structure between the two species could be used to predict population structure. In addition, comparative assessments of the population genetic structure of sympatric species can provide valuable information about the factors that influence population structuring (Avise, 2010). For example, environmental deterioration and intensive fishery exploitation has threatened the abundance of *X. boulengeri* and *X. nudicorpa* (Cao, 2003). Previous studies have shown that the abundance of *X. boulengeri* decreased following the construction of a dam, which directly reduced the available habitat (Wang et al., 2012). Habitat loss can cripple the ability of fish to respond to environmental changes. Previous studies have shown that genetic homogeneity appears in endemic fish with limited habitat in the upper reaches of the Yangtze River (Liu et al., 2017; Shen et al., 2017a). To the best of our knowledge, genetic diversity and population structure data have not previously been reported for *X. boulengeri* and *X. nudicorpa*. Nuclear genes and mitochondrial genes have been widely used in population genetic studies (Sun et al., 2013; Buonaccorsi et al., 2012; Gao et al., 2016; Ibrahim et al., 2010; Ramey, 1995; Grijalva-Chon et al., 1994; Domingues et al., 2018; Domínguez-Contreras et al., 2018). The combination of microsatellite DNA and mitochondrial markers can be a good method to corroborate genetic diversity and structure (Förschler et al., 2009).

In the present study, microsatellite DNA and mitochondrial DNA (mtDNA) markers were used to investigate the patterns of population genetic structure for *X. boulengeri* and *X. nudicorpa*. Simple sequence repeat (SSR) data, jointly with traditional summary statistics including *F*_ST_ and *F*_is_, were used to assess the population genetic structure by Structure analysis. The Cytochrome *b* (Cyt *b*, protein coding gene in mtDNA), control region (CR, regulator region in mtDNA) sequences were then used to examine these patterns through time to detect demographic history. We aimed to: (i) compare genetic diversity and population genetic structures between *X. boulengeri* and *X. nudicorpa*; (ii) compare the occurrence of recent population expansions between the two species in order to assess demographic history; and (iii) clarify spatial genetic sub-structuring as a good base for improving *Xenophysogobio* stock management.

## Materials and Methods

### Ethics statement

All handling of *X. boulengeri* and *X. nudicorpa* specimens was conducted in strict accordance with Animal Experimental Ethical Inspection of Laboratory Animal Centre, Yangtze River Fisheries Research Institute, Chinese Academy of Fishery Sciences (ID Number: FRE0006).

### Sample collection and experimental methods

A total of 227 *X. boulengeri* individuals from five sites, Jiangjin (JJ), YB, Shuifu (SF), Yongshan (YS), and Qianwei (QW), and 126 *X. nudicorpa* individuals from four sites, JJ, YB, QJ and PZH, were collected between 2011 and 2018 (Table 1; Fig. 1). All samples were identified based on morphological characteristics (Ding, 1994), and small fins were clipped and preserved in 99% ethanol for DNA extraction.

Genomic DNA was isolated by proteinase *K* digestion followed by a salt extraction method (Aljanabi & Martinez, 1997). Nine polymorphic microsatellite primers (Xb2, Xb3, Xb4, Xb5, Xb11, LT-C5, LT-D2, LT-D7, LT-D8) in *X. boulengeri* and nine polymorphic microsatellite primers (LT-C5, LT-C6, LT-C7, LT-D1, LT-D2, LT-D3, LT-D7, LT-D8, LT-D9) in *X. nudicorpa* were used, according to previously reported protocols (Cheng et al., 2012; Zeng et al., 2015). The final products were used for SSR analysis based on capillary electrophoresis fluorescence on the ABI 377 DNA Analyzer (Tianyihuiyuan Bio-Technology Co., Ltd, Wuhan, China), and the results were analyzed by the GeneMarker 1.5 software (Hulce et al., 2011). The Cyt *b* gene was amplified by polymerase chain reaction (PCR) using primers L14724: (5′-GACTTGAAAAACCACCGTTG-3′) and H15915 (5′-CTCCGATCTCCGGATTACAAG-3′) (Xiao, Zhang & Liu, 2001), and CR using MitDI-F (5′-CACCCYTRRCTCCCAAAGCYA-3′) and MitDI-R (5′-GGTGCGGRKACTT GCATGTRTAA-3′) (Shen et al., 2017a). The PCR amplification was carried out in 50 μl volumes containing 25 μl of mix (Qingke Biological Technology Co., Ltd Wuhan, China), 2 μl of template DNA, 2 μl of each primer (10 mM/l), and 19 μl of ultrapure water. The PCR amplification conditions were performed by first denaturation step at 94 °C for 3 min, followed by 35 cycles at 94 °C for 30 s, at 55 °C for 30 s, and at 72 °C for 30 s, plus a final extension at 72 °C for 8 min. PCR products were sequenced in both directions with the same primers as PCR in an ABI 3730XL sequencer (Tianyihuiyuan Bio-Technology Co., Ltd, Wuhan, China).

Latitude, longitude and altitude were measured by a hand-held sub meter GPS (GEO-XT6000; Nanjing Jun can Instrument Co., Ltd., Nanjing, China).

### Statistical analysis of microsatellite data

Genetic diversity indexes of microsatellite loci were calculated and genetic diversity parameters, including the number of alleles, number of effective alleles, observed heterozygosity (Ho) and expected heterozygosity (He) were all detected by POPGENE version 1.32 (Yeh & Boyle, 1997). Duncan’s multiple comparison procedure was used to compare the means for the genetic diversity values between Jinsha River populations (PZH and QJ) and Yangtze River populations (JJ and YB) in *X. nudicorpa* by SPSS 17.0. The analysis of molecular variance (AMOVA) and pairwise *F*_ST_ based microsatellite data were assessed in Arlequin version 3.1.1 (Excoffier, Laval & Schneider, 2005). Benjamini Hochberg was applied to correct the *P*-values of *F*_ST_ whenever multiple tests were performed. Due to the long distance and the geographic isolation of two dams between Jinsha River populations and Yangtze River populations, we divided *X. nudicorpa* populations into two groups: Group 1 represented the Jinsha River populations (PZH and QJ), and Group 2 represented the Yangtze River populations (JJ and YB). The global *F*_ST_ was also calculated between Group 1 and Group 2. Correlations between geographical distances (ln km) and genetic distances (*F*_ST_/(1-*F*_ST_)) based on microsatellite data were tested in GENALEX version 6.5 (Peakall & Smouse, 2012). Geographic distances following the river network were measured in Google Earth (© 2018 Google) (Lisle, 2006). POPGENE was also performed to detect deviations from the Hardy–Weinberg equilibrium (HWE). The inbreeding coefficient (*F*_is_) and allelic richness were obtained by FSTAT version 2.9.3 (Goudet, 1995) and the null alleles were checked by the software Micro-Checker version 2.2.3 (Oosterhout et al., 2004).

Genetic structure analyses of populations identified using the microsatellite loci were conducted. We examined genetic relationships among populations in Structure version 2.3.4 (Pritchard, Stephens & Donnelly, 2000). Structure applies a Bayesian framework to identify the most likely number of cluster in the sample. The delta *K* method was used to determine the appropriate value of *K* (Evanno, Regnaut & Goudet, 2005). The lengths of the Markov chain Monte Carlo (MCMC) reps after burnin were set to 1,200,000 with a burn-in period of 200,000. The simulated *K* values ranged from 1 to 10, and 10 independent runs were used for each *K* with correlated allelic and without locprior (Wilson et al., 2015). The most likely *K* value was chosen according to the peak value of the mean log likelihood [Ln *P*(*X*/*K*)] and the Delta *K* statistic for a given *K*. The results were summarized on the online platform Structure Harvester (http://taylor0.biology.ucla.edu/struct_harvest/). Genetic relationships among populations were also examined by applying discriminant analysis of principal components (DAPC) (Jombart, Devillard & Balloux, 2010) using the adegenet 2.0.1 package in R 3.2.2 software (R Development Core Team, 2014). For microsatellite data, CONVERT version 1.3 (Glaubitz, 2004) was used to transform the input formats of the following programs: POPGENE, ARLEQUIN, and STRUCTURE.

### Statistical analysis of mtDNA sequences

Sequences were aligned using the Clustal X program (Thompson et al., 1997) and pruned using the software MEGA version 6.06 (Tamura et al., 2013). Genetic diversity indexes of mtDNA sequences were calculated. The haplotype (*h*) and nucleotide (π) diversity, the number of haplotypes and the haplotype frequencies were obtained using the software Dnasp version 5.10 (Librado & Rozas, 2009). To estimate population differentiation, global *F*_ST_ and pairwise *F*_ST_ were calculated using Arlequin version 3.1.1 (Excoffier, Laval & Schneider, 2005). The global *F*_ST_ was also calculated between Group 1 and Group 2 based on Cyt *b* and CR datasets. To test for signatures of isolation by distance, correlations between geographical distances (ln km) and genetic distances (*F*_ST_/(1-*F*_ST_)) were tested using a MANTEL matrix correlation test in GENALEX version 6.5 (Peakall & Smouse, 2012).

Genetic structure analyses of populations identified using the mtDNA sequences were conducted. To visualize intraspecific genetic variation, median-joining networks of haplotypes were constructed in the software NETWORK version 5.0.0.3 (Bandelt, Forster & Röhl, 1999). Phylogenetic analyses were also conducted. Before reconstructing the phylogenetic trees, optimal *X. boulengeri* and *X. nudicorpa* DNA substitution models (TN+F+I; HKY+F+I and HKY+F+G4; HKY+F+I, respectively) were obtained for Cyt *b* and CR sequences using the Bayesian information criterion in IQ-TREE (Nguyen et al., 2015). *X. boulengeri* and *X. nudicorpa* are sister taxon, and they were used as outgroups each other. We downloaded *X. boulengeri* and *X. nudicorpa* mtDNA sequences from Genbank to perform phylogenetic analyses (AF375868, KM516103, KM373519, KM255691, NC-025300, KU314698). Bayesian inference was performed to reconstruct the phylogenetic tree among the Cyt *b* and CR haplotypes in *X. boulengeri* and *X. nudicorpa* in MRBAYES version 3.1.2 (Ronquist et al., 2012). Divergence times among the detected mitochondrial clades were evaluated in BEAST version 1.8.0 (Bouckaert et al., 2014) using an uncorrelated relaxed molecular clock Bayesian approach, in addition to a Yule prior approach and a random starting tree. The mean mutation rate was specified as a normal distribution, and estimates were calibrated using two age constraints. One constraint represented an upper bound of 5.3 Ma, the divergence time of *Hemibarbus barbus* (AB070241) and *Hemibarbus labeo* (DQ347953) or *Gnathopogon elongates* (AB218687) and *Gobiocypris rarus* (NC_018099) based on fossils from the Late Miocene (Zhou, 1990). The second time constraint represented a lower bound of 1.1 Ma, derived from the Kunlun-Yellow River tectonic movement (KP316067, NC_033403, AB239595), which occurred before this time (Cui et al., 1998; He & Chen, 2007). The MCMC chain was run for 1 × 10^7^ generations and was sampled every 10,000 generations. The first 25% were burn-in. TRACER version 1.5 (Goderniaux et al., 2009) was used to test the convergence of the chains to the stationary distribution, which was determined by an effective sample size (ESS) of more than 200. Trees were visualized in interactive tree of life (Ivica & Peer, 2016). The values of transition/transversion (Ts/Tv) were calculated in MEGA version 6.06 (Tamura et al., 2013). Automated barcode gap detection (ABGD) was used for species delimitation using the online version (http://wwwabi.snv.jussieu.fr/public/abgd/abgdweb.html) (Eberle, Warnock & Ahrens, 2016). The first significant gap in the distribution of sequence distances beyond intraspecific sequence divergence can thus be used to infer operational taxonomic units that may be related to species. One critical parameter of the ABGD method is the prior maximum divergence of intraspecific diversity (*P*). Default settings were used for the prior range for maximum intraspecific divergence (0.001, 0.1), corrected distances (JC69) and minimum slope increase (*X*) of 1.5. Although there is still a lack of consensus of how to interpret discordant ABGD results (Kekkonen & Hebert, 2014), previous studies advocate using a *P*-value of ∼0.01, which has previously been demonstrated to recover species accurately (Eberle, Warnock & Ahrens, 2016; Puillandre et al., 2012).

Tajima’s *D* (Tajima, 1989) and Fu’s *F*s (Fu & Li, 1993) statistics were examined to infer the neutrality deviant. The mismatch distribution was also performed to evaluate the demographic history in populations of *X. boulengeri* and *X. nudicorpa*. Neutrality tests and Mismatch distribution were obtained in Dnasp. The Bayesian skyline plot method was used to infer the population dynamics of *X. boulengeri* and *X. nudicorpa*. After sequences were calculated in BEAST version 2.4.3 (Bouckaert et al., 2014), the chain convergence and the skyline plot graphic were visualized in Tracer version 1.5 (Goderniaux et al., 2009) with ESS of more than 200. In addition, Neutrality tests, Mismatch distribution and Bayesian skyline plot were also carried out separately for samples of *X. nudicorpa* between the Jinsha River and the Yangtze River because of a significant difference in genetic structure between these locations.

## Results

### Population genetic diversity

A total of 271 *X. boulengeri* individuals from five sites and 164 *X. nudicorpa* individuals from four sites were screened across nine microsatellite loci, respectively. For *X. boulengeri*, the Ho and He per population varied from 0.766 (JJ) to 0.825 (SF) and from 0.852 (JJ) to 0.879 (SF), respectively, (Table 2). Moreover, the global level of genetic diversity for *X. nudicorpa* was moderately lower than for that of *X. boulengeri.* For *X. nudicorpa*, the Ho and He per population varied from 0.482 (QJ) to 0.613 (JJ) and from 0.469 (PZH) to 0.705 (YB), respectively, (Table 2). In sympatric populations (JJ and YB), the level of genetic diversity was similar for *X. boulengeri* and *X. nudicorpa* (Table 2).

**Table 2:** The genetic diversity levels based on mtDNA and SSR.

	MtDNA (Cyt *b*)				MtDNA (CR)				nuclear DNA (SSR)						
	*N*	*H*	*h*	π	*N*	*H*	*h*	π	*N*	NA	Ne	Ar	Ho	He	*F*_i__s_
JJ															
*X. boulengeri*	55	32	0.963	0.00373	37	12	0.614	0.00194	66	16.2	10.9	8.346	0.766	0.852	0.102
*X. nudicorpa*	54	34	0.951	0.00388	63	23	0.768	0.00302	60	13.2	8.9	3.855	0.613	0.696	0.121
YB															
*X. boulengeri*	128	61	0.959	0.00404	112	24	0.787	0.00175	72	16.8	10.7	8.224	0.792	0.863	0.083
*X. nudicorpa*	5	4	0.900	0.00372	5	3	0.800	0.00281	11	6.2	5.2	3.943	0.593	0.705	0.174
SF															
*X. boulengeri*	7	7	1.000	0.00297	7	7	1.000	0.00292	14	8.7	7.1	8.667	0.825	0.879	0.066
YS															
*X. boulengeri*	22	21	0.996	0.00485	23	9	0.779	0.00194	45	14.6	10.2	8.362	0.785	0.872	0.102
QW															
*X. boulengeri*	15	10	0.914	0.00454	46	17	0.852	0.00237	74	17.8	11.2	8.323	0.817	0.869	0.061
QJ															
*X. nudicorpa*	3	1	0.000	0.00000	3	2	0.667	0.00078	6	2.9	2.6	2.889	0.482	0.474	−0.020
PZH															
*X. nudicorpa*	44	3	0.132	0.00014	55	3	0.261	0.00032	87	6.6	3.6	2.708	0.483	0.469	−0.030
Total															
*X. boulengeri*	227	92	0.963	0.00405	225	43	0.791	0.00201	271						
*X. nudicorpa*	106	37	0.718	0.00348	126	23	0.752	0.00304	164						

**Note:**

Mitochondrial genetic diversity levels based Cyt *b* and CR: mtDNA sample size = *N*, number of haplotypes = *H*, haplotype diversity = *h*, nucleotide diversity = π. SSR genetic diversity across nine (*Xenophysogobio boulengeri*) and nine (*Xenophysogobio nudicorpa*) loci in *Xenophysogobio boulengeri* and *Xenophysogobio nudicorpa*, respectively: microsatellite sample size = *N*, number of alleles = NA, number of effective alleles = Ne, allelic richness = Ar, observed heterozygosity = Ho, expected heterozygosity = He, inbreeding coefficient = *F*_is_.

We obtained 994 bp and 967 bp sequences (after being aligned) for Cyt *b* in 227 individuals of *X. boulengeri* and 106 individuals of *X. nudicorpa*, respectively. For both species, the overall Ts/Tv ratios were 41.014 and 339.736, respectively. A total of 92 *X. boulengeri* haplotype and 37 *X. nudicorpa* haplotype sequences were deposited in GenBank (accession numbers MK001561–MK001652, MK001696–MK001732). Overall, haplotype and nucleotide diversities were high for *X. boulengeri* (*h* = 0.963, π = 0.00405), but lower for *X. nudicorpa* (*h* = 0.718, π = 0.00348) (Table 2). Within the same population (JJ and YB), overall genetic diversities were similar for both species.

Control region sequences amplified from 225 *X. boulengeri* individuals and 126 *X. nudicorpa* individuals were distributed into 43 (784 bp fragments) and 23 haplotypes (854 bp fragments), respectively, (Table 2). These sequences were deposited in GenBank (accession numbers MK001653–MK001695, MK001733–MK001755). The CR sequences had 39 and 18 variable sites for *X. boulengeri* and *X. nudicorpa*, respectively. The overall Ts/Tv ratio was 7.908 in *X. boulengeri* and 1.273 in *X. nudicorpa*. The global haplotype and nucleotide diversities were similar for both *X. boulengeri* (*h* = 0.791, π = 0.00201) and *X. nudicorpa* (*h* = 0.752, π = 0.00304) (Table 2). Compared to the mtDNA Cyt *b* marker, the values of haplotype and nucleotide diversities based on the mtDNA CR were lower in samples from all populations among *X. boulengeri* and *X. nudicorpa* (excluding PZH and QJ populations) (Table 2).

In terms of single species, five *X. boulengeri* populations appeared similar in genetic diversity. However, there was a difference in genetic diversity among *X. nudicorpa* populations. Duncan’s test shown that the genetic diversity in Jinsha river populations were significantly lower than that in Yangtze River populations (Table 2; Table S1).

For SSR dataset, 16 out of 50 HWE tests (32%) displayed a departure in *X. boulengeri* (Table S2). For *X. nudicorpa*, five out of 30 (17%) deviated from HWE (Table S3). Estimates of inbreeding coefficients showed that the values were positive in all populations of *X. boulengeri* (0.061–0.102) and JJ and YB populations of *X. nudicorpa* (0.121 and 0.174), except the values were negative in QJ and PZH populations of *X. nudicorpa* (Table 2).

### Population genetic structure

For mtDNA and SSR dataset, AMOVA analysis showed that weak signal of geographic structure was detected in *X. boulengeri* (SSR, *F*_ST_ = 0.0003, *P* = 0.71359; Cyt *b*, *F*_ST_ = 0.0047, *P* = 0.22092; CR, *F*_ST_ = 0.0352, *P* = 0.00196) (Table 3). High geographic structure was detected in comparison to *X. boulengeri*. A substantial proportion of genetic variation was related to differences among groups (SSR, 8.85%; Cyt *b*, 47.45%; CR, 44.03%), with highly significant *F*_ST_ values (SSR, *F*_ST_ = 0.0612, *P* < 0.0001; Cyt *b*, *F*_ST_ = 0.5291, *P* < 0.0001; CR, *F*_ST_ = 0.5792, *P* < 0.0001) (Wright, 1978) (Table 3).

**Table 3:** Analysis of molecular variance (AMOVA) of geographical populations for *Xenophysogobio boulengeri* and *Xenophysogobio nudicorpa*.

		Source of variation	Variance components	Percentage of variation	*F* statistics	*P*	Tajima’s *D*	Fu’s *F*s
*X. boulengeri*	Cyt *b*	Among populations	0.00951	0.47	0.0047	0.22092	−2.154**	−123.271**
		Within populations	2.00855	99.53				
		Total	2.01806					
	Cyt *b*-Clade	Among populations	0.59666	44.28	0.4428**	<0.0001		
		Within populations	0.75091	55.72				
		Total	1.34757					
	CR	Among populations	0.02805	3.52	0.0352**	0.00196	−2.269**	−51.676**
		Within populations	0.76811	96.48				
		Total	0.79616					
	SSR	Among populations	0.00035	0.03	0.0003	0.71359		
		Within populations	1.24845	99.97				
		Total	1.24880					
*X. nudicorpa*	Cyt *b*	Among groups	1.08089	47.45	0.5291**	<0.0001	−2.045*	−27.240**
		Among populations within groups	0.12437	5.46	0.1039*	0.03812	−1.308^J^	−2.235^J^
		Within populations	1.07274	47.09	0.4745	0.36852	−2.127^Y^*	−31.066^Y^**
		Total	2.27800					
	CR	Among groups	0.79461	44.03	0.5792**	<0.0001	−0.939	−9.635**
		Among populations within groups	0.25055	13.88	0.2481*	0.01173	−0.591^J^	−0.659^J^
		Within populations	0.75938	42.08	0.4403	0.34702	−1.191^Y^	−13.289^Y^**
		Total	1.80454					
	SSR	Among groups	0.03931	8.85	0.0612**	<0.0001		
		Among populations within groups	−0.01213	−2.73	−0.0300	0.90518		
		Within populations	0.41720	93.88	0.0885	0.31085		
		Total	0.44438					

**Note:**

The Cyt *b*-Clade represent the analysis between Clade I and II based on Cyt *b* for *Xenophysogobio boulengeri*; Jinsha River populations are denoted with a ^J^ and Yangtze River populations are denoted with a ^Y^. Statistically significant estimations (*P* < 0.01; *P* < 0.05) are denoted with a ** and a *.

Among geographic populations within species, genetic differentiation was estimated by pairwise *F*_ST_. The pairwise *F*_ST_ were lower than 0.05 and significant in few cases for *X. boulengeri* (Wright, 1978) (Table 4), which indicated weak signal gene differentiation among the five *X. boulengeri* populations. In contrast, a modest divergence was observed by pairwise *F*_ST_ results between JJ and PZH in *X. nudicorpa*. For *X. nudicorpa*, the pairwise *F*_ST_ values revealed significant differentiation between the Jinsha River (PZH and QJ) and Yangtze River (JJ and YB) populations (Table 5). For mtDNA dataset, the values of pairwise *F*_ST_ are larger than that of SSR dataset.

**Table 4:** Pairwise estimates of genetic differentiation (below) and *P*-values (above) of *Xenophysogobio boulengeri*.

	JJ	YB	SF	YS	QW
JJ					
Cyt *b*		0.685	0.270	0.621	0.198
CR		<0.001	<0.001	0.309	<0.001
SSR		0.919	0.919	0.919	0.919
YB					
Cyt *b*	−0.005		0.198	0.621	0.198
CR	0.060**		<0.001	0.613	0.613
SSR	0.006		0.919	0.919	0.919
SF					
Cyt *b*	0.030	0.037		0.198	0.198
CR	0.228**	0.136**		0.018	0.150
SSR	−0.010	−0.002		0.919	0.919
YS					
Cyt *b*	−0.003	−0.006	0.067		0.621
CR	0.013	−0.007	0.140*		0.613
SSR	−0.001	0.002	−0.013		0.919
QW					
Cyt *b*	0.030	0.019	0.121	−0.017	
CR	0.068**	−0.002	0.076	−0.005	
SSR	−0.003	0.004	−0.011	0.000	

**Note:**

Statistically significant estimations (*P* < 0.01; *P* < 0.05) are denoted with a ** and a *.

**Table 5:** Pairwise estimates of genetic differentiation (below) and *P*-values (above) of *Xenophysogobio nudicorpa*.

	JJ	YB	QJ	PZH
JJ				
Cyt *b*		0.068	0.054	<0.001
CR		<0.001	0.041	<0.001
SSR		0.937	0.432	<0.001
YB				
Cyt *b*	0.110		0.530	<0.001
CR	0.244**		0.441	<0.001
SSR	−0.029		0.937	0.324
QJ				
Cyt *b*	0.357	0.048		0.991
CR	0.400*	0.067		0.441
SSR	−0.001	−0.040		0.932
PZH				
Cyt *b*	0.550**	0.625**	−0.188	
CR	0.598**	0.556**	0.006	
SSR	0.070**	0.040	−0.030	

**Note:**

Statistically significant estimations (*P* < 0.01; *P* < 0.05) are denoted with a ** and a *.

**Figure 2:**
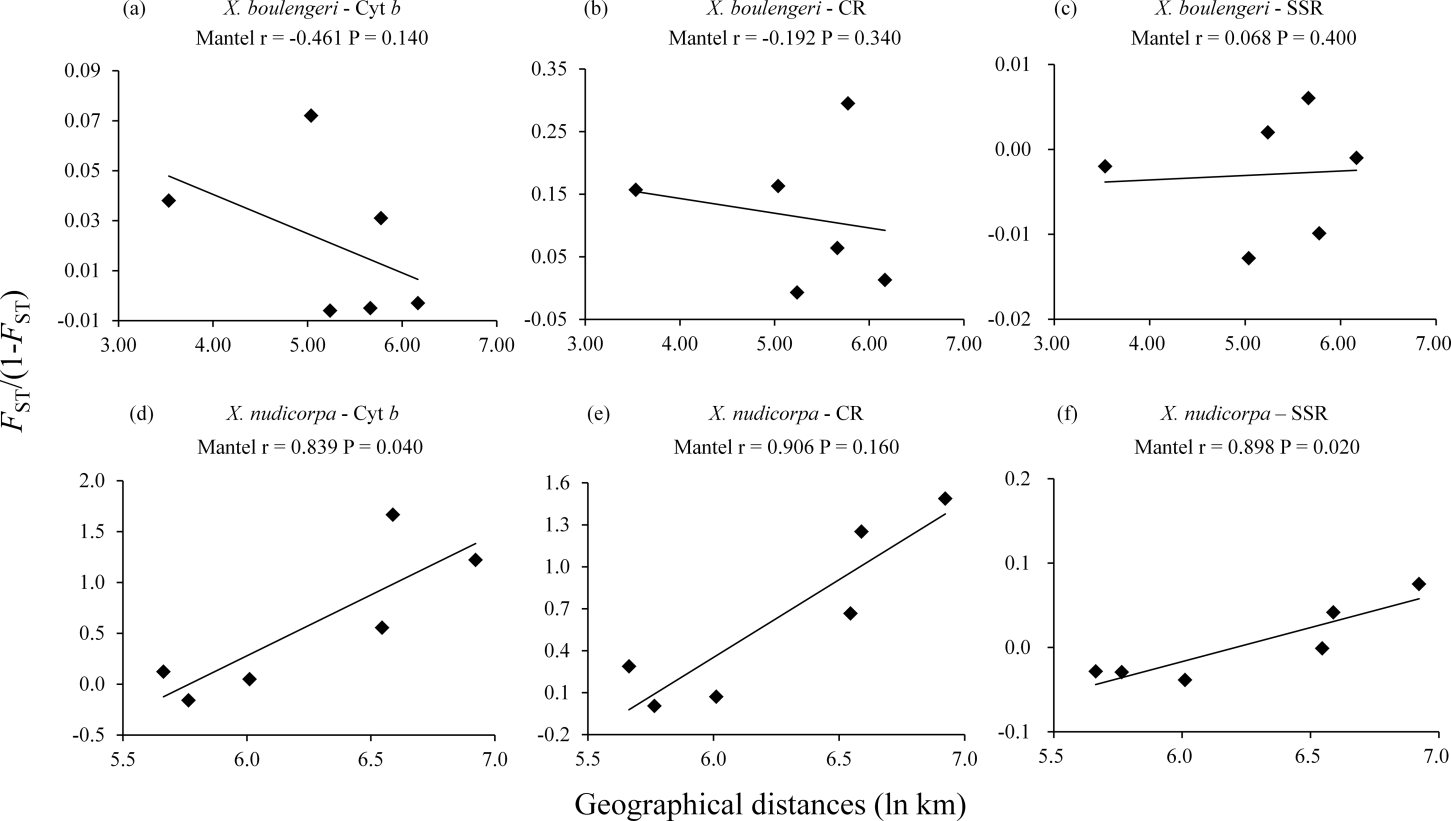
Isolation by distance analysis for pairwise population comparisons in *X. boulengeri* and *X. nudicorpa*, where *F*_ST_/(1-*F*_ST_) was regressed over the geographical distance (ln km). (A–C) *X. boulengeri* based on Cyt *b*, CR and SSR, (D–F) *X. nudicorpa* based on Cyt *b*, CR and SSR.

No correlation were detected between geographical distances (ln km) and genetic distances (*F*_ST_/(1−*F*_ST_)) for all the three markers dataset in *X. boulengeri*. For *X. nudicorpa*, a significant correlation was detected in the SSR (*r* = 0.898, *P* = 0.020) and Cyt *b* dataset (*r* = 0.839, *P* = 0.040), but not in CR (*r* = 0.906, *P* = 0.160) (Fig. 2; Table S4 and S5).

**Figure 3:**
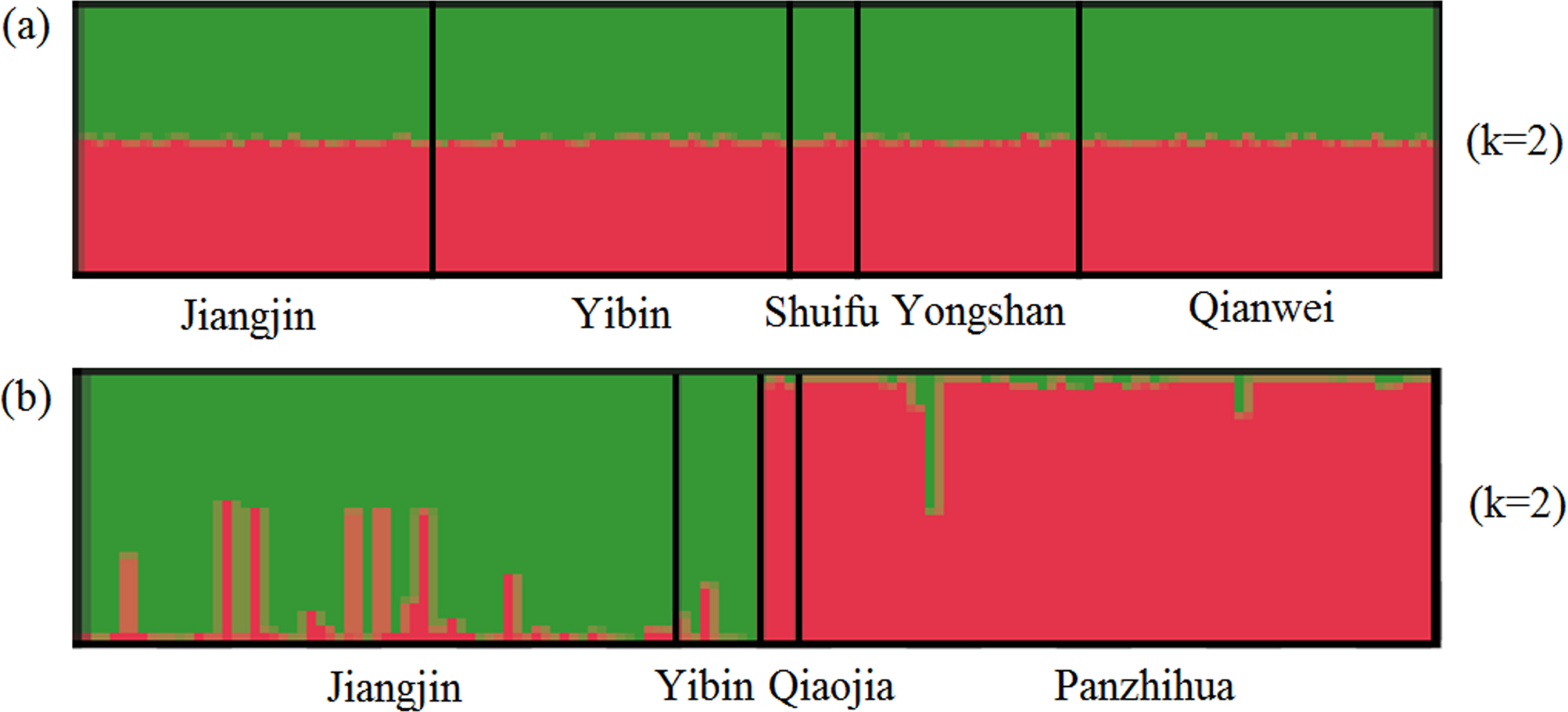
Structure clustering conducted based on microsatellite loci within populations of (A) *X. boulengeri* and (B) *X. nudicorpa*. Structure results at *K* = 2, with different colors indicating different clusters.

For SSR dataset, a structure analysis was also used to detect population structure. For *X. boulengeri* and *X. nudicorpa*, the Delta *K* values were highest (8.02 and 667.01, respectively) when *K* = 2 (Fig. S1). The structure analysis indicated greater differences among both the Jinsha and Yangtze River populations in *X. nudicorpa*, but not in *X. boulengeri* (Figs. 3A and 3B), supporting the results of the global differentiation. The DAPC clusters showed obvious clusters between the Jinsha and Yangtze River populations of *X. nudicorpa* (Fig. S2B), but not in *X. boulengeri* (Fig. S2A).

**Figure 4:**
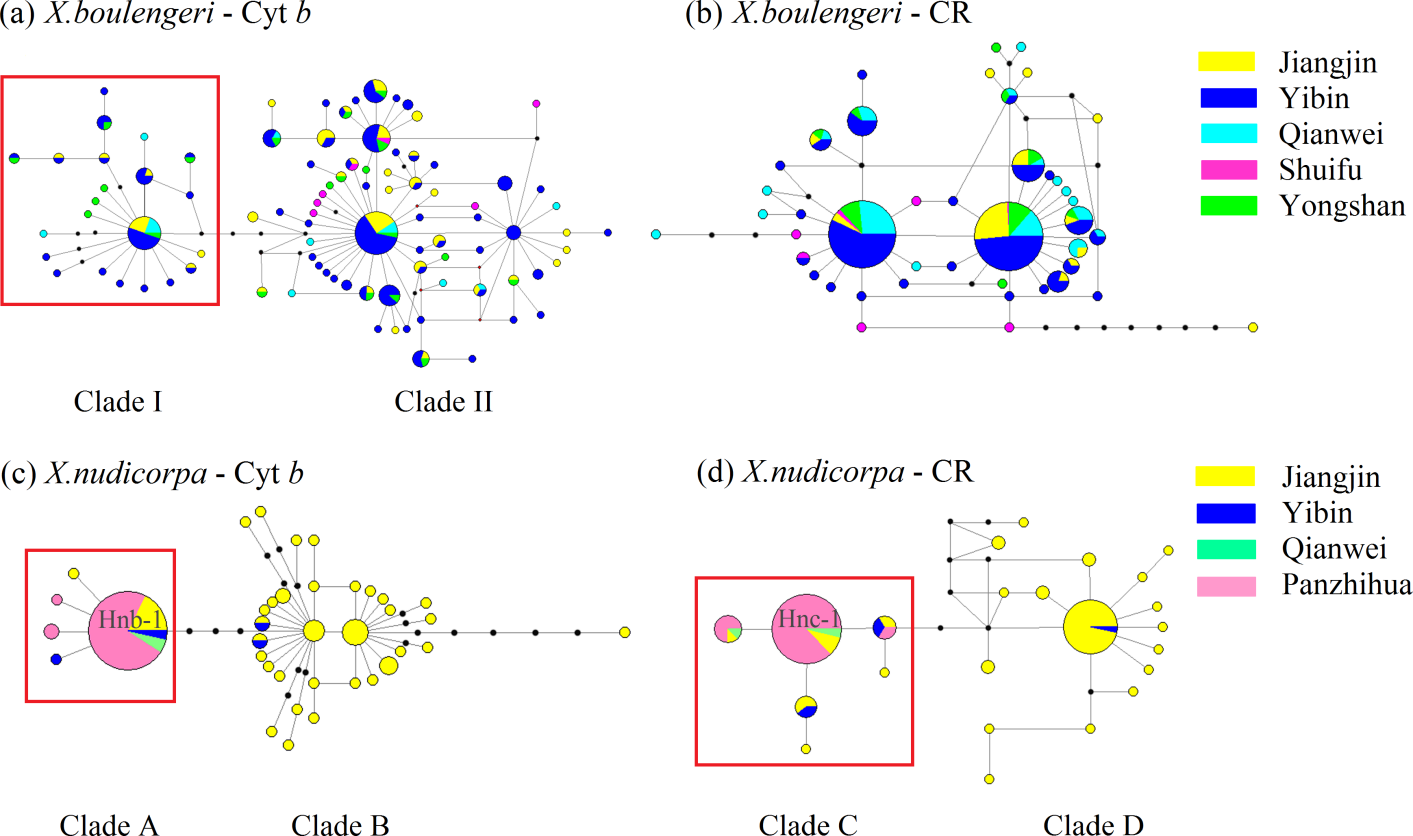
Haplotype network (based on neighboring-join) of *X. boulengeri* based on (A) Cyt *b* and (B) CR and *X. nudicorpa* based on (C) Cyt *b* and (D) CR. Each circle represents a haplotype and their size is proportional to their frequencies. The colors represent the geographic origin of haplotypes, as indicated in the legend. Hnb-1 stands for H-1 based on Cyt *b* for *X. nudicorpa* in Fig. 4C, and “Hnc-1” stands for H-1 based on CR for *X. nudicorpa* in Fig. 4D.

For mtDNA dataset, the haplotype network was performed to detected population structure. The network presented a star shape, dominated by four common haplotypes for the Cyt *b* dataset and two common haplotypes for the CR dataset, with no geographic aggregation for haplotypes in *X. boulengeri* (Figs. 4A and 4B). The Cyt *b* haplotypes of *X. boulengeri* were divided into two clades (Clade I and Clade II) in network topology. There were five mutational steps between clades (Fig. 4A). Based on CR data, clades could not be observed in the haplotypes network of *X. boulengeri* (Fig. 4B). The AMOVA analysis also corroborated the divergence between Clade I and Clade II with a high and significant *F*_ST_ value (*F*_ST_ = 0.4428, *P* < 0.0001) (Table 3).

For *X. nudicorpa*, the results of haplotype network showed two clades based on Cyt *b* (Clade A and Clade B) and CR (Clade C and Clade D) respectively. Clade A and Clade C encompassed individuals from four populations with the most frequent haplotype usually being present in the Jinsha River populations (PZH and QJ), whereas Clade B and Clade D individuals were found only in JJ and YB (Figs. 4C and 4D). For PZH population, 41 out of 44 (93%) and 47 out of 55 (85%) individuals were located in Hnb-1 (Fig. 4C) and Hnc-1 (Fig. 4D), respectively.

**Figure 5:**
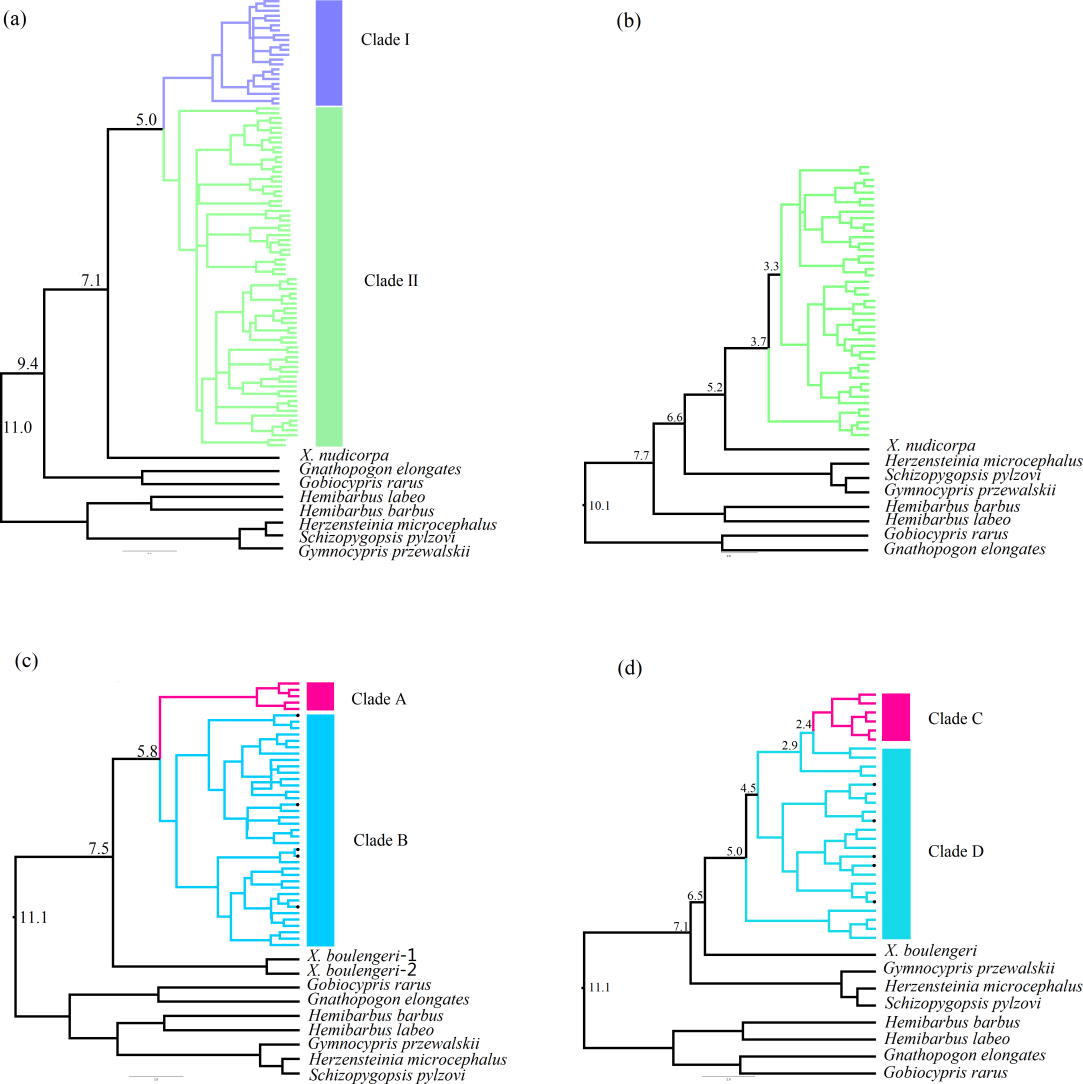
Divergence time estimation with time-calibrated points was reconstructed in *X. boulengeri* based on (A) Cyt *b* and (B) CR and *X. nudicorpa* based on (C) Cyt *b* and (D) CR. Numbers up branches indicated the time of species divergence events occurred (Ma: million years ago). The black dots on the branches represent the sequences downloaded from GenBank.

The Bayesian inference phylogenetic trees supported the monophyly of Clade I, Clade A and Clade C (Figs. S3A, S3C and S3D), which were consistent with the haplotype networks. Divergence time between Clade I and Clade II was 5.0 Ma from the Cyt *b* sequence in *X. boulengeri* (Fig. 5A). For *X. nudicorpa*, the Cyt *b* and CR datasets showed that divergence time was not consistent in clades, 5.8 Ma between Clade A and Clade B from Cyt *b* sequence and 2.4 Ma between Clade C and Clade D from the CR sequence (Figs. 5C and 5D).

Further species delimitation analysis has been performed using ABGD based on the Cyt *b* and CR datasets, which suggested one species based on recursive partitioning over a range of prior values for maximum intraspecific divergence in *X. boulengeri* (Puillandre et al., 2012), as well as *X. nudicorpa*, when *P*-value is closest to 0.01 (Table S6). For all Cyt *b* or CR sequences of *X. boulengeri* and *X. nudicorpa*, ABGD analysis suggested a total of two species (Table S6), which indicated that no new species has been formed.

### Past demographic history

Neutrality tests were used to detect population expansions using Cyt *b* and CR sequences. For *X. boulengeri* and *X. nudicorpa*, Tajima’s *D* and Fu’s *F*s were significant with negative values (except Tajima’s *D* for the CR dataset in *X. nudicorpa*) (Table 3), which indicated demographic expansion events. Due to genetic divergence, neutrality tests (Tajima’s *D* and Fu’s *F*s) for the Jinsha River and Yangtze River *X. nudicorpa* populations were calculated separately. The values of Tajima’s *D* and Fu’s *F*s were significant and negative in Yangtze River populations (except Tajima’s *D* for the CR dataset), but not significant in Jinsha River populations.

**Figure 6:**
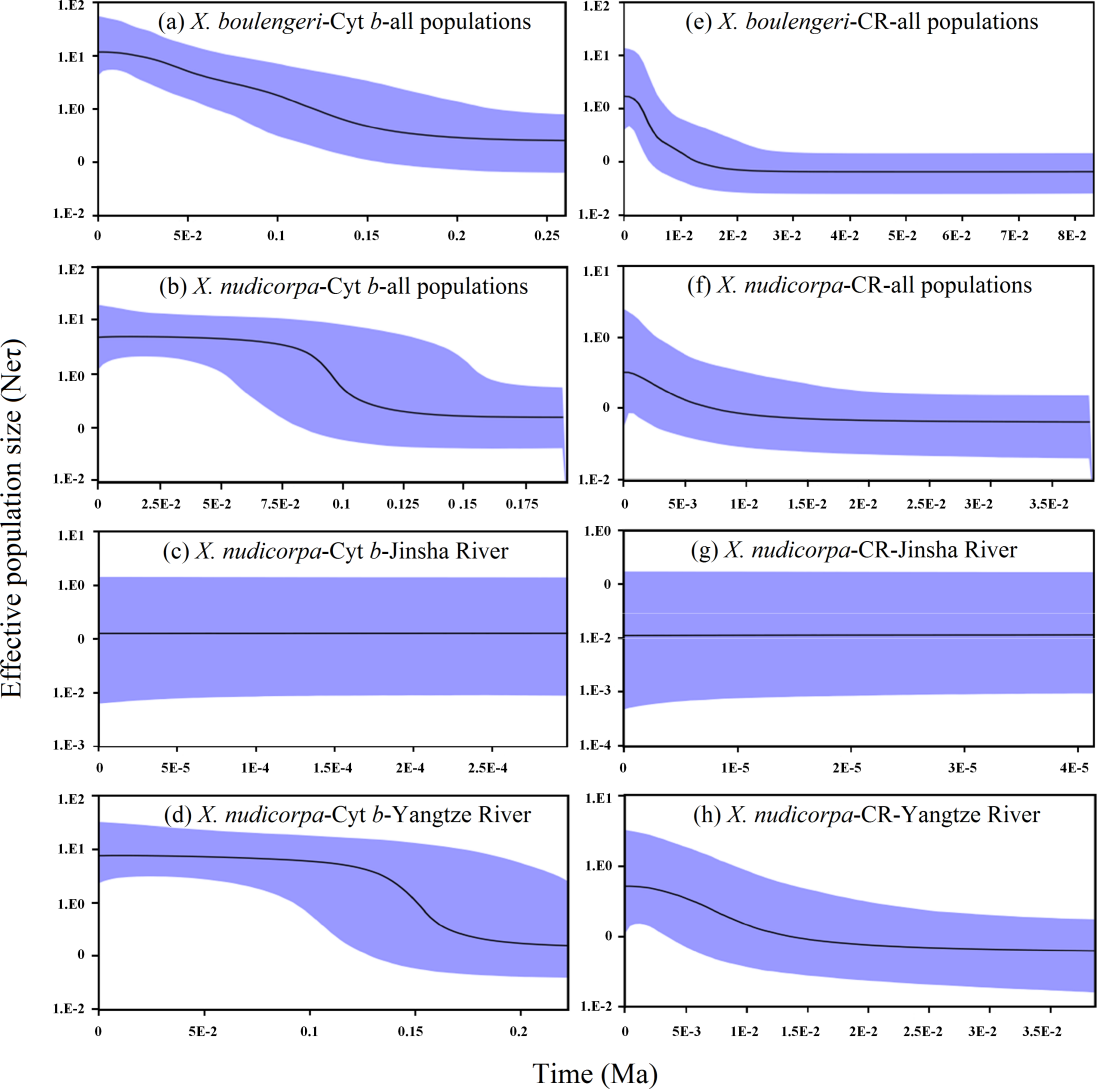
Graphs of extended Bayesian Skyline Plot for *X. boulengeri* and *X. nudicorpa* based on Cyt *b* and CR. (A–D) For Cyt *b* dataset: (A) *X. boulengeri* from all populations, (B) *X. nudicorpa* from all populations, (C) *X. nudicorpa* from Jinsha River, (D) *X. nudicorpa* from Yangtze River, (E–H) For CR dataset, (E) *X. boulengeri* from all populations, (F) *X. nudicorpa* from all populations, (G) *X. nudicorpa* from Jinsha River, (H) *X. nudicorpa* from Yangtze River. Ne represents the effective population size, τ represents generational time of the organism, the black line depicts the median population size, and the shaded areas represented the 95% confidence intervals of HPD analysis.

A mismatch distribution and Bayesian skyline plot indicated recent demographic expansion events in *X. boulengeri* and *X. nudicorpa* based on the Cyt *b* and CR datasets. For *X. boulengeri*, a smooth unimodal mismatch distribution was observed, which was compatible with a single expansion (Rogers & Harpending, 1992). *X. nudicorpa* showed a bimodal distribution pattern. Jinsha River populations had narrow mismatch distributions that differed from those of the Yangtze River populations, although the mismatch distribution graphs were both unimodal (Fig. S4). Mismatch distributions were very conservative for detecting population growth (Ramos-Onsins & Rozas, 2002). A unimodal mismatch distribution, consisting of only two types of pairwise differences in Jinsha River populations of *X. nudicorpa*, failed to indicate a recent population expansion. Growth in population size according for Bayesian skyline plot suggested demographic expansion among populations in *X. boulengeri* and *X. nudicorpa* (Figs. 6A and 6B). However, we found Jinsha River populations remained at a stable population size (Fig. 6C), while Yangtze River populations had increased (Fig. 6D).

Taking into account a substitution rate of 0.65% per million years in the Cyt *b* gene of mtDNA (Macey et al., 1998), this episode of demographic expansion of *X. boulengeri* and *X. nudicorpa* began separately approximately 20–200 Ka (Fig. 6A) and 75–135 Ka (Fig. 6B), respectively. The Jinsha River *X. nudicorpa* populations had a stable population size between approximately 0–0.5 Ka (Fig. 6C), and the Yangtze River *X. nudicorpa* populations had an increasing population size between approximately 125–175 Ka (Fig. 6D).

For the CR dataset, demographic expansions in Yangtze River *X. boulengeri* and *X. nudicorpa* populations were consistent with the Cyt *b* dataset. Taking into account a substitution rate of 3.6% per million years in the CR of mtDNA (Donaldson & Wilson, 1999), these episodes of demographic expansion began separately in *X. boulengeri* and *X. nudicorpa* approximately 1–20 Ka (Fig. 6E) and 1–10 Ka (Fig. 6F), respectively. The Jinsha River *X. nudicorpa* populations remained at a stable size between approximately 0–0.04 Ka (Fig. 6G), and the Yangtze River *X. nudicorpa* populations had an increasing population size between approximately 2–15 Ka (Fig. 6H).

## Discussion

### Population genetic diversity

Genetic diversity forms the basis for species to respond to natural selection (Vrijenhoek, 1994). In the case of mtDNA and SSR markers, the genetic diversity (*h* and π) of *X. nudicorpa* was similar to *X. boulengeri* in JJ and YB populations (Table 2), indicating similar natural selection pressure in sympatric habitats for both species. The upper reaches of the Yangtze River, as the origin center of Gobiobotinae fish, is a sympatric habitat for these two species. In sympatric habitats, similar natural selection pressures can result in similar genetic variation, which can contribute to similar levels of genetic diversity.

For *X. boulengeri*, the level of genetic diversity was similar among populations, which was consistent with the short distances and absence of any long-term geographical barriers among these five populations. However, different levels of genetic diversity were detected between the Jinsha and Yangtze River *X. nudicorpa* populations. The low level of genetic diversity in Jinsha River populations (PZH and QJ) might have been caused by the founder effect or genetic drift (Franklin & Frankham, 1998). In addition, *X. boulengeri* was only rarely present in the QJ-PZH section of the Jinsha River. In contrast, the abundance of *X. nudicorpa* in the Jinsha River was higher than that in the Yangtze River (Gao et al., 2013). We surmise that *X. nudicorpa*, particularly individuals with the most frequent mtDNA haplotype, is better adapted to the local environment. However, low genetic diversity would reduce the capacity to cope with environmental change in the Jinsha River populations of *X. nudicorpa* (Franklin & Frankham, 1998).

Interestingly, the genetic diversity of the Cyt *b* gene was higher than that of the CR in these two species, except for *X. nudicorpa* populations in the Jinsha River (Table 2). The ratio of Ts/Tv was also lower in the CR than in the coding Cyt *b*. This phenomenon is not unique to *X. boulengeri* and *X. nudicorpa*, although most species have higher rates of mutation in CR than in Cyt *b*. Low rates of evolution in the CR have been reported in salmonids (Shedlock et al., 1992), *Melanotaenia* (Zhu et al., 1994) and *Saurogobio* (Zhang, 2001).

### Population genetic structure

In sympatric habitats, the genetic structure of *X. boulengeri* and *X. nudicorpa* followed similar patterns, with weak signal of geographic population structure. Extrinsic factors, such as historical vicariant events and the same environmental features, can promote the development of similar patterns of population structure (McMillen-Jackson & Bert, 2003) observed for *X. boulengeri* and *X. nudicorpa*. Moreover, similar spawning patterns and migration behaviors for both species likely also contributed to the similar population structures.

For *X. boulengeri*, genetic homogeneity among populations indicated high levels of gene flow (Nm) (Table S7), which might be related to the habit of spawning drifting eggs (Liu, Zhou & Zhou, 2012). In addition, similar environment features, such as low altitude and a subtropical climate (except the YS which had a temperate climate) (Table 1), may have some influence on the genetic structure in *X. boulengeri* (Zhou et al., 2016). The genetic homogeneity among populations of *X. boulengeri* was congruent with genetic structure reports in other taxa from these regions (Liu et al., 2017; Shen et al., 2017a; Liu, Zhou & Zhou, 2012).

For *X. nudicorpa*, a significant correlation between genetic and geographic distance in Mantel’s test was observed using the Cyt *b* and SSR dataset, although the result was not supported by the CR datasets. Hence, population genetic differentiation cannot be explained by the isolation-by-distance model using the present data. The lack of strong evidence in the CR (Fig. 2) further suggests that the present pattern was shaped by a historical connection rather than ongoing gene flow (Hubert et al., 2007). We also cannot conclude that dams have affected the genetic structure of the two fish species. The reasons are that: (1) populations of *X. boulengeri* above and below the Xiangjiaba Dam did not show distinct divergence; and (2) accumulation of mutational steps between the two clades of *X. nudicorpa*, corresponding to either side of the dams, need a longer period of time than that lapsed since dam construction (2007 for the Xiluodu Dam and 2008 for the Xiangjiaba Dam). Thus, the current genetic patterns of the fish are likely to be derived from historical events. The Jinsha River was insulated in the middle Pliocene (Clark et al., 2004), which was congruent with the divergence time between the Jinsha and Yangtze River populations (5.8−2.4 Ma). Hence, geographic structure might be related to long-term isolation between the Jinsha and Yangtze River in the middle Pliocene. The climate and environmental heterogeneity between the Jinsha and Yangtze Rivers might also be the factors that lead to differences in genetic diversity and significant genetic structure between the fish populations of the two rivers (Zhou et al., 2016). The Yangtze River is characterized by a typical subtropical climate and the Jinsha River by a temperate climate (QJ) or three-dimensional climate based on the south subtropical zone (PZH) (Table 1).

Hardy–Weinberg Departure (HWD) was mostly due to excess of homozygosity. The micro-checker detected excess of homozygosity for most HWD tests (Table S8 and S9). The positive values of *F*_is_, indicating heterozygote deficit, might be due to the Wahlund effect caused by the subpopulation structure (Dharmarajan, Beatty & Rhodes, 2013). If the sample includes a mixture of individuals from more than one breeding unit, then (on average) Ho will be less than He (Waples, 2015). The phylogenetic trees showed the genetic divergence within populations with low support rate based Cyt *b* in *X. boulengeri*. In addition, inbreeding behavior might also be one of the reasons for heterozygote deficit. Population size of *X. boulengeri* and *X. nudicorpa* were decreased due to environmental deterioration and intensive fishery exploitation. Moreover, the presence of null alleles may one cause of heterozygote deficit.

The concept of management unit (MU) is useful for identifying and prioritizing conservation units within a species. MUs are recognized as populations with significant divergence of allele frequencies at mitochondrial or nuclear loci, regardless of the phylogenetic distinctiveness of the alleles (Huang et al., 2007; Moritz, 1994). According to the definitions above, a single MU was identified within *X. boulengeri*, and two separate MUs within *X. nudicorpa* including Jinsha River Unit and Yangtze River Unit.

### Past demographic history

The inferences about demographic history based on Cyt *b* and CR provide evidence of a recent population expansion in *X. boulengeri* and *X. nudicorpa* derived from the Yangtze River. However, the population expansion times obtained by Cyt *b* and the CR gene were not coincident. The interval might have been caused by the substitution rates of the markers we used. The average substitution rates used in the present study were those of vertebrate mtDNA (0.65% for Cyt *b* and 3.6% for CR). However, variation of the CR was lower than that of Cyt *b* in this study, against the common property of vertebrate mtDNA, and therefore the time deduced from the CR may be underestimated. For both *X. boulengeri* and the Yangtze River populations of *X. nudicorpa*, the beginnings of the expansion took place in a similar period in the Late Pleistocene. *X. boulengeri* has undergone a greater long-term expansion period than Yangtze River *X. nudicorpa* populations. Population expansion for both species has provided evidence that the main stream in the upper reaches of the Yangtze River could have been a refuge for *X. boulengeri* and *X. nudicorpa* in the Late Pleistocene. Similar population expansion events might contribute to the similar genetic structure pattern found in *X. boulengeri* and *X. nudicorpa* (Silva et al., 2018). Any expansion events might be related to past climatological changes and geological events (Huang et al., 2007). In the Late Pleistocene, a warm climate (Avise, Walker & Johns, 1998) and fluctuating water levels (Yang, 1986) provided population expansion chances for *X. boulengeri* and the Yangtze River populations of *X. nudicorpa.* The effects of a warming postglacial climate upon the demography of many species have been well documented in the upper reaches of the Yangtze River Basin, including *Leptobotia microphthalma* (Shen et al., 2017a), *L. elongate* (Liu, Zhou & Zhou, 2012), *L. rubrilabris* (Shen et al., 2017b) and *Coreius heterodon* (Yuan et al., 2010).

Conversely, the Bayesian skyline plot suggested that Jinsha River populations of *X. nudicorpa* were stable, and no recent population expansion has occurred. Stable populations should present a lower number of recently evolved mutations than expanding populations (Templeton, 2006). The low frequency of recent mutations, singletons or doubletons, in the Jinsha River populations of *X. nudicorpa* supports the hypothesis of a stable population, which is consistent with the neutrality tests and Bayesian skyline plot. Most likely, this genetic pattern reflects the distinct demographic history traits of *X. nudicorpa* between the Jinsha and Yangtze River populations, which can be related to differences in genetic diversity. In Yangtze River, the Pleistocene refuges might have contributed to the colonization for *X. nudicorpa* by creating opportunities for suitable establishment in stable areas during the climatic fluctuations of the Pleistocene. More individuals could survive and propagate to increase population size and the level of genetic diversity (Hubert et al., 2007).

Divergence time between Jinsha and Yangtze River populations could provide evidence for geographic events. In the middle Pliocene, the Jinsha River was insulated from the Yangtze River (Clark et al., 2004). During the Late Pliocene (≤3.4 Ma), the uplift of the eastern Qinghai-Tibetan Plateau resulted in river capture events, and drainage rearrangements occurred. The Jinsha River flowed into the Yangtze River, and the modern drainage basin morphology was formed (Clark et al., 2004). The two population genetic groups of *X. nudicorpa* corresponds to the geography of the past rather than modern drainage systems, and likely results from the insulated Jinsha River in the middle Pliocene, and associated river captures (Zhang, Comes & Sun, 2011). The divergence time between the Jinsha and Yangtze River populations falls into the middle Pliocene. This timing broadly agrees with both geological and molecular data, indicating that geographic isolation occurred between the Jinsha and Yangtze Rivers during the middle Pliocene. The similar genetic divergence has been reported in the Euchiloglanis fish complex (Li, Ludwig & Peng, 2017).

## Conclusions

In sympatric habitats, the genetic diversity and structure of *X. boulengeri* and *X. nudicorpa* were similar. Specifically, in the upper reaches of the Yangtze River, *X. boulengeri* and *X. nudicorpa* experienced population expansion events, whereas the Jinsha River populations of *X. nudicorpa* did not. Similar genetic diversity and structure might be due to similar life history, ecology and trophic characteristics. Using spatial genetic sub-structuring, we detected no geographic population divergence in *X. boulengeri*. For *X. nudicorpa*, we detected obvious geographic population divergence. Genetic homogeneity in *X. boulengeri* might be related to similar population expansion events and environmental features. Significant geographic genetic subdivision in *X. nudicorpa* might have been caused by the geographic isolation in the middle Pliocene, as well as the climate and environmental heterogeneity between the Jinsha and Yangtze Rivers, but not for the dams.

Based on the present study regarding genetic diversity and structure, several management suggestions can be raised. In sympatric habitats, similar genetic diversity and structure of *X. boulengeri* and *X. nudicorpa* were detected. However, the abundance of *X. nudicorpa* was obviously lower than *X. boulengeri*. Hence, first it would be valuable to further explore why *X. nudicorpa* was in a disadvantaged state in the same region, by investigating factors such as reproductive strategy, parasitic infection and environmental adaptability. Second, *X. boulengeri* and *X. nudicorpa* both experienced population expansion events and represented high levels of genetic diversity in the upper reaches of the Yangtze River. These sections might provide key habitat and be a refuge for *X. boulengeri* and *X. nudicorpa*. Therefore, a greater level of protection should be implemented in this region. In particular, a fishing ban should be introduced to reduce fishing pressure and channel regulation should be avoided as far as possible to protect the available habitat for *X. boulengeri* and *X. nudicorpa*, as well as other benthic fish. Third, *X. boulengeri* populations appear a single MU, and population connectivity should be guaranteed. In these sections, new dams should not be constructed, and river connectivity should be maintained. In addition, greater attention should be paid to the YS populations due to the barrier of a dam in this area. For *X. nudicorpa*, two MUs deserve separate conservation attention. Fourth, the Jinsha River *X. nudicorpa* populations were disadvantageous to response to environmental changes because of low genetic diversity and genetic drift, and further exploration of the adaptive mechanisms to the local environment is needed. Key adaptations to environmental factors should be explored, and corresponding management strategies should be proposed to maintain genetic diversity if possible. Finally, small population sizes might lead to inbreeding behaviour, artificial breeding could be considered to increase population sizes for both species, especially *X. nudicorpa*. In addition, further research is required to explore the environmental factors as well as invasive species influencing genetic diversity.

## Supplemental Information

10.7717/peerj.7393/supp-1 Supplemental Information 1 The line chart among Delta K and K. Delta K as a function of the K values according to 10 run outputs. (a) *Xenophysogobio boulengeri* of five populations, (b) *Xenophysogobio nudicorpa* of four populations.

10.7717/peerj.7393/supp-2 Supplemental Information 2 Results of a discriminant analysis of principal components (DAPC), showing relationships among geographic population clusters. (a) *Xenophysogobio boulengeri* based on nine microsatellite loci and (b) *Xenophysogobio nudicorpa* based on nine microsatellite loci.

10.7717/peerj.7393/supp-3 Supplemental Information 3 Bayesian inference (BI) phylogenetic tree based on haplotypes. (**a-b**) *Xenophysogobio boulengeri* based on Cyt *b* and CR, (**c**-**d**) *Xenophysogobio nudicorpa* based on Cyt *b* and CR. Numbers represented nodal supports inferred. The supported value was only displayed among main clades.

10.7717/peerj.7393/supp-4 Supplemental Information 4 Observed (bars) and expected (lines) mismatch distribution according to the recent expansion model of *X.boulengeri* and (b) *X.nudicorpa* populations. (a) *Xenophysogobio boulengeri* from all populations based on Cyt *b*, (b) *Xenophysogobio boulengeri* from all populations based on CR; (c-e) *Xenophysogobio nudicorpa* from all populations, Jinsha River and Yangtze River based on Cyt *b*, (f-h) *Xenophysogobio nudicorpa* from all populations, Jinsha River and Yangtze River based on CR. XB and XN are the abbreviation of *Xenophysogobio boulengeri* and *Xenophysogobio nudicorpa* respectively.

10.7717/peerj.7393/supp-5 Supplemental Information 5 Significance test of the genetic diversity index between Jinsha River populations and Yangtze River populations in *Xenophysogobio nudicorpa.*. Statistically significant estimations (*p* < 0.05) are denoted with a*.

10.7717/peerj.7393/supp-6 Supplemental Information 6 P values of Chi square test for all the loci in Xenophysogobio boulengeri. The values in bold displayed a departure from HWE.

10.7717/peerj.7393/supp-7 Supplemental Information 7 P values of Chi square test for all the loci in Xenophysogobio nudicorpa. The values in bold displayed a departure from HWE.

10.7717/peerj.7393/supp-8 Supplemental Information 8 Genetic distance among populations of *Xenophysogobio boulengeri.*. Genetic distance has been calculated by the formula: *F*_ST_/(1-*F*_ST_).

10.7717/peerj.7393/supp-9 Supplemental Information 9 Genetic distance among populations of *Xenophysogobio nudicorpa.*. Genetic distance has been calculated by the formula: *F*_ST_/(1-*F*_ST_).

10.7717/peerj.7393/supp-10 Supplemental Information 10 Species delimitation for *Xenophysogobio boulengeri* and *Xenophysogobio nudicorpa* based on Cyt *b* and CR using Automated Barcode Gap Detection (ABGD).

10.7717/peerj.7393/supp-11 Supplemental Information 11 Nm among populations of *Xenophysogobio boulengeri* (*below*) *and* *Xenophysogobio nudicorpa* (*above*).

10.7717/peerj.7393/supp-12 Supplemental Information 12 Microchecker null allele results for *Xenophysogobio boulengeri.*. Yes indicates the presence of an allele, No indicates the absence of an allele,–indicates insuffient sample.

10.7717/peerj.7393/supp-13 Supplemental Information 13 Microchecker null allele results for Xenophysogobio nudicorpa. Yes indicates the presence of an allele, No indicates the absence of an allele,–indicates insuffient sample.

10.7717/peerj.7393/supp-14 Supplemental Information 14 SSR data of *Xenophysogobio boulengeri.*. Raw data of *Xenophysogobio boulengeri* from five populations based 9 microsatellite loci..

10.7717/peerj.7393/supp-15 Supplemental Information 15 SSR data of *X.nudicorpa*. Raw data of *Xenophysogobio nudicorpa* from four populations based on 9 microsatellite loci.

10.7717/peerj.7393/supp-16 Supplemental Information 16 CR data of *Xenophysogobio boulengeri*.. Raw data of *Xenophysogobio boulengeri* from five populations based CR data.

10.7717/peerj.7393/supp-17 Supplemental Information 17 Cyt *b* data of *Xenophysogobio boulengeri*.. Raw data of *Xenophysogobio boulengeri* from five populations based Cyt *b* data.

10.7717/peerj.7393/supp-18 Supplemental Information 18 CR data of *X.nudicorpa*.. Raw data of *Xenophysogobio nudicorpa* from four populations based CR data.

10.7717/peerj.7393/supp-19 Supplemental Information 19 Cyt *b* data of *X.nudicorpa*.. Raw data of *Xenophysogobio nudicorpa* from four populations based Cyt *b* data.

## Additional Information and Declarations

### Data Availability

The following information was supplied regarding data availability: Haplotype sequences of mtDNA are available in GenBank, accession number: MK001561–MK001755. The raw data of microsatellite DNA are available in the Supplemental Files (Table S10 and S11).
