# Supplementary figures and images for: Genetic structure of two sympatric gudgeon fishes (Xenophysogobio boulengeri and X. nudicorpa) in the upper reaches of Yangtze River Basin
Source: PeerJ. 2019 Aug 6;7:e7393. doi: 10.7717/peerj.7393 (PMC6688597; doi:10.7717/peerj.7393)

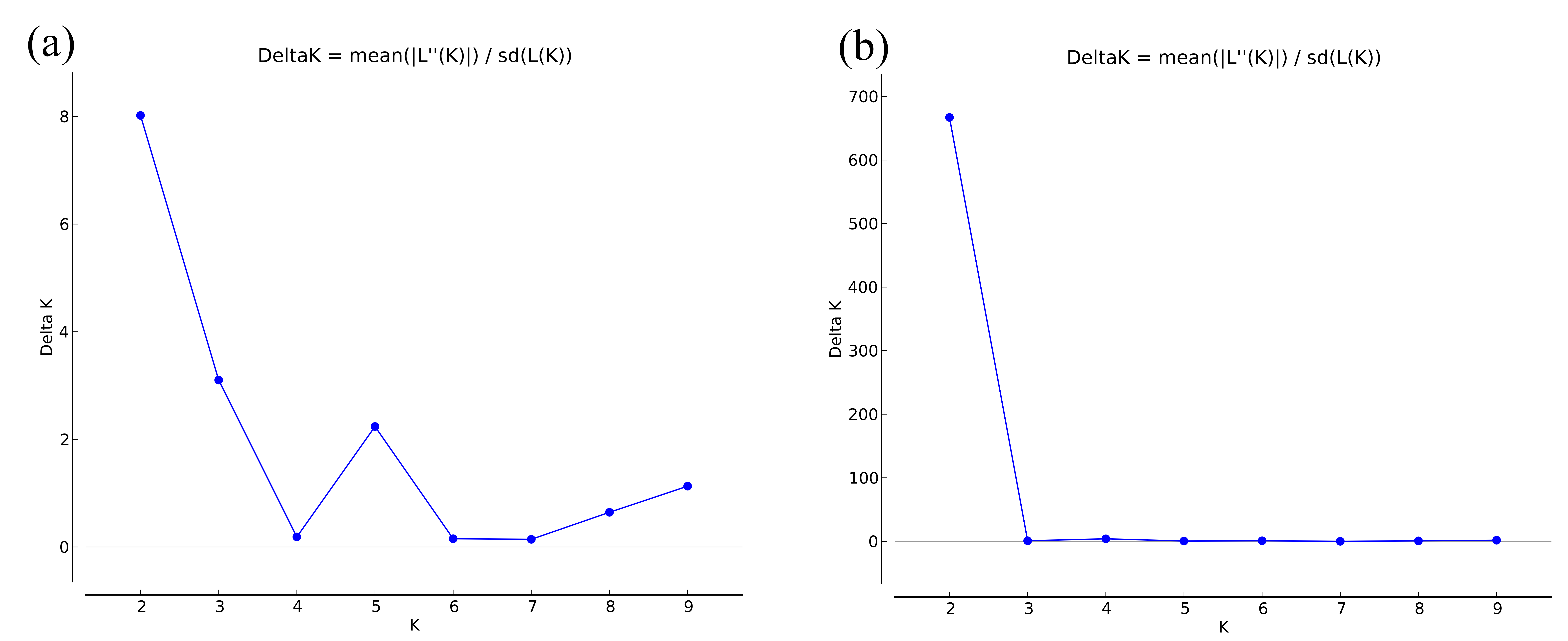

Supplement: Supplemental Information 1 — Delta K as a function of the K values according to 10 run outputs. (a) Xenophysogobio boulengeri of five populations, (b) Xenophysogobio nudicorpa of four populations. [file peerj-07-7393-s001.png]

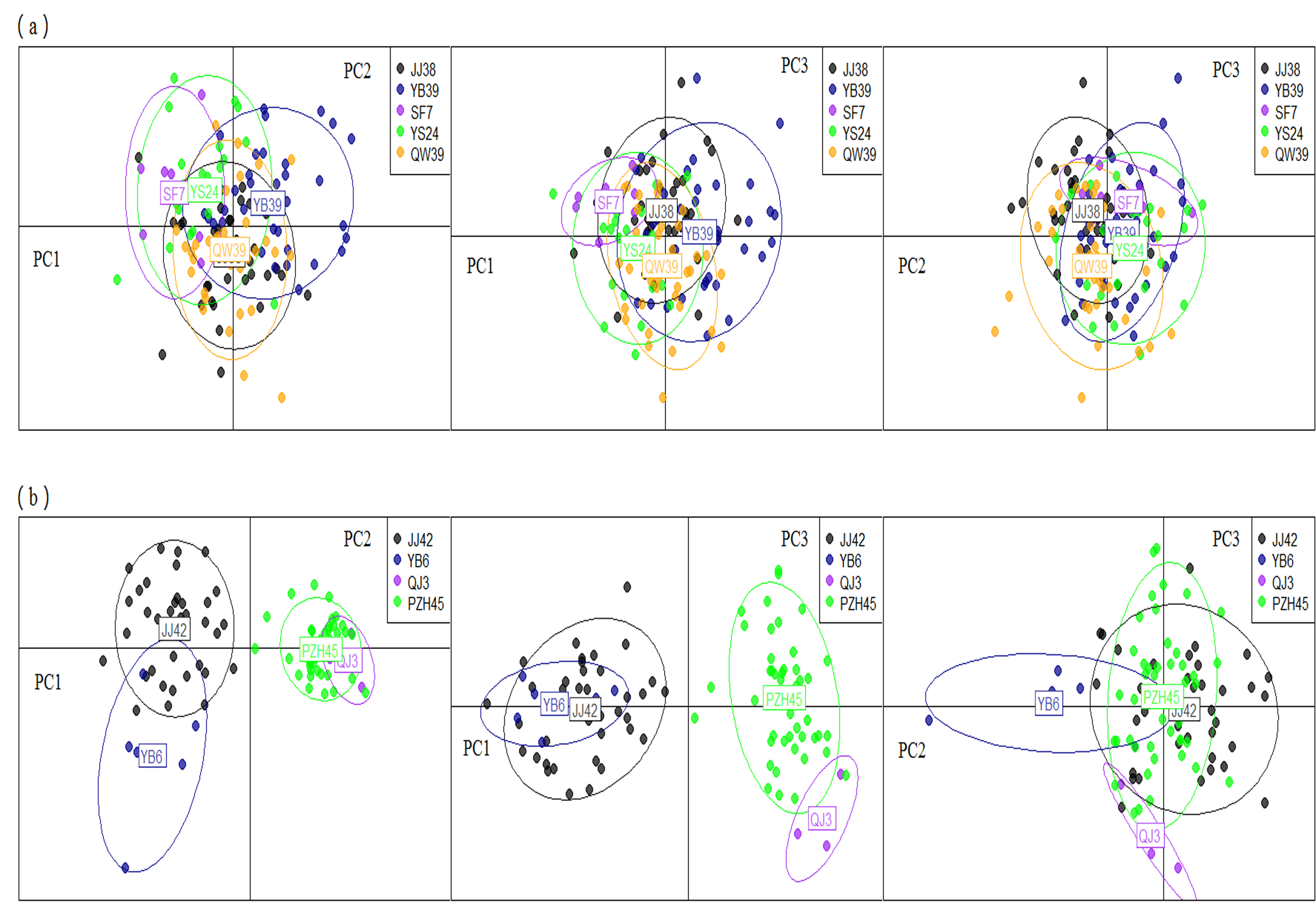

Supplement: Supplemental Information 2 — (a) Xenophysogobio boulengeri based on nine microsatellite loci and (b) Xenophysogobio nudicorpa based on nine microsatellite loci. [file peerj-07-7393-s002.png]

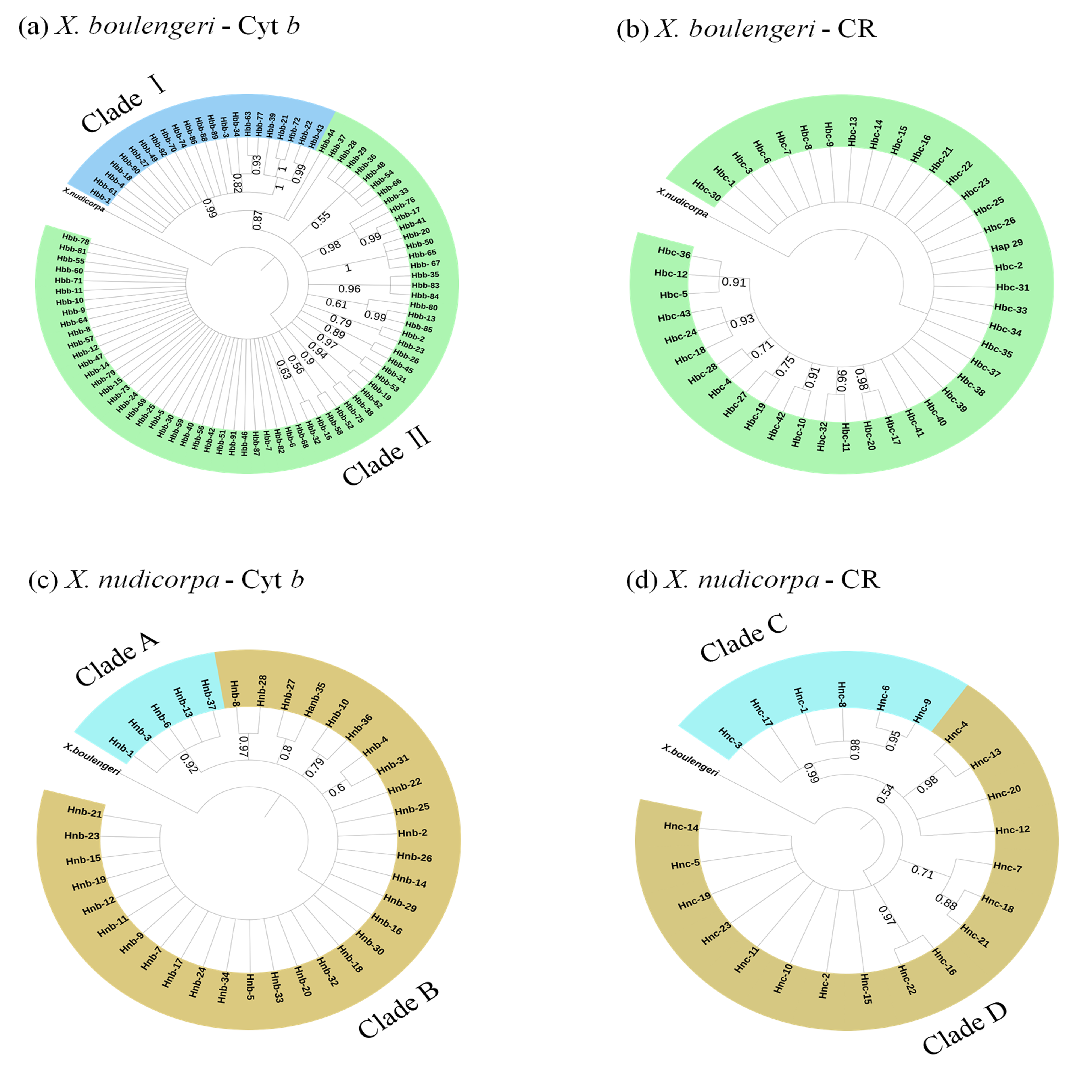

Supplement: Supplemental Information 3 — (a-b) Xenophysogobio boulengeri based on Cyt b and CR, (c-d) Xenophysogobio nudicorpa based on Cyt b and CR. Numbers represented nodal supports inferred. The supported value was only displayed among main clades. [file peerj-07-7393-s003.png]

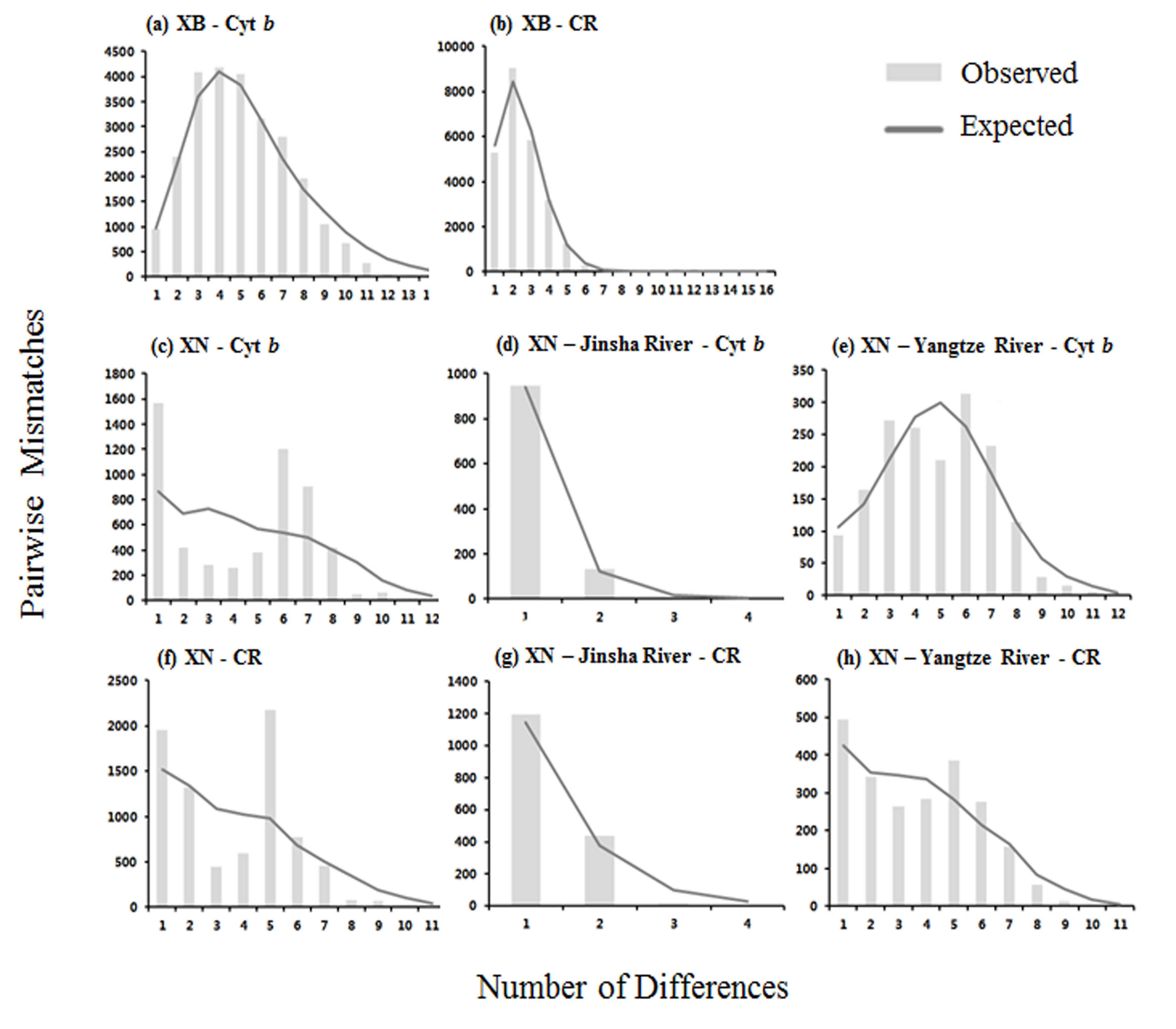

Supplement: Supplemental Information 4 — (a) Xenophysogobio boulengeri from all populations based on Cyt b, (b) Xenophysogobio boulengeri from all populations based on CR; (c-e) Xenophysogobio nudicorpa from all populations, Jinsha River and Yangtze River based on Cyt b, (f-h) Xenophysogobio nudicorpa from all populations, Jinsha River and Yangtze River based on CR. XB and XN are the abbreviation of Xenophysogobio boulengeri and Xenophysogobio nudicorpa respectively. [file peerj-07-7393-s004.png]
