# Supplementary material for: Genetic structure of two sympatric gudgeon fishes (Xenophysogobio boulengeri and X. nudicorpa) in the upper reaches of Yangtze River Basin
Source: PeerJ. 2019 Aug 6;7:e7393. doi: 10.7717/peerj.7393 (PMC6688597; doi:10.7717/peerj.7393)
Supplement: Supplemental Information 5 — Statistically significant estimations (p < 0.05) are denoted with a*. [file peerj-07-7393-s005.docx]

|  | Genetic diversity index | Jinsha River | Yangtze River | *P* value |
| --- | --- | --- | --- | --- |
| MtDNA (Cyt b) | *h* | 0.00007 ± 0.00009 | 0.92250 ± 0.03606 | 0.001* |
|  | π | 0.00007 ± 0.00009 | 0.00380 ± 0.00011 | 0.001* |
| MtDNA (CR) | *h* | 0.46400 ± 0.28708 | 0.78400 ± 0.02262 | 0.257 |
|  | π | 0.47150 ± 0.00353 | 0.70050 ± 0.00636 | 0.001* |
| nuclear DNA (SSR) | *H*_O_ | 0.48250 ± 0.00070 | 0.60300 ± 0.01414 | 0.007* |
|  | *H*_E_ | 0.47150 ± 0.00353 | 0.70050 ± 0.00636 | 0.001* |
