# Supplementary material for: Genetic structure of two sympatric gudgeon fishes (Xenophysogobio boulengeri and X. nudicorpa) in the upper reaches of Yangtze River Basin
Source: PeerJ. 2019 Aug 6;7:e7393. doi: 10.7717/peerj.7393 (PMC6688597; doi:10.7717/peerj.7393)
Supplement: Supplemental Information 6 — The values in bold displayed a departure from HWE. [file peerj-07-7393-s006.docx]

|  | **LT-C5** | **LT-D2** | **LT-D7** | **LT-D8** | **Xb3** | **Xb2** | **Xb4** | **Xb11** | **Xb5** |
| --- | --- | --- | --- | --- | --- | --- | --- | --- | --- |
| JJ | 0.90 | 0.52 | 0.13 | **0.00** | **0.01** | 0.09 | 0.89 | **0.00** | **0.00** |
| YB | 0.79 | 0.66 | **0.02** | 0.44 | 0.47 | **0.00** | **0.04** | 0.72 | **0.01** |
| SF | 0.85 | 0.45 | 0.45 | 0.64 | 0.71 | 0.94 | 0.35 | 0.87 | 0.31 |
| YS | 0.94 | **0.01** | **0.00** | 1.00 | 0.33 | 0.79 | 0.82 | **0.03** | 0.16 |
| QW | **0.00** | **0.01** | 0.06 | 0.15 | **0.00** | 1.00 | 0.87 | **0.00** | **0.00** |
