# Supplementary material for: Genetic structure of two sympatric gudgeon fishes (Xenophysogobio boulengeri and X. nudicorpa) in the upper reaches of Yangtze River Basin
Source: PeerJ. 2019 Aug 6;7:e7393. doi: 10.7717/peerj.7393 (PMC6688597; doi:10.7717/peerj.7393)
Supplement: Supplemental Information 7 — The values in bold displayed a departure from HWE. [file peerj-07-7393-s007.docx]

|  | **LT-C5** | **LT-C6** | **LT-C7** | **LT-D1** | **LT-D2** | **LT-D3** | **LT-D7** | **LT-D8** | **LT-D9** |
| --- | --- | --- | --- | --- | --- | --- | --- | --- | --- |
| JJ | **0.00** | 0.07 | 0.99 | **0.01** | 0.49 | 0.92 | 0.25 | **0.00** | 1.00 |
| YB | **0.01** | 0.99 | 0.08 | 0.38 | 0.38 | 0.27 | 0.76 | 0.07 |  |
| QJ |  |  |  | 0.68 | 0.15 | 0.92 | 0.73 | 0.92 |  |
| PZH | 1.00 |  | 1.00 | 0.61 | 0.87 | 0.27 | 0.93 | **0.00** | 1.00 |
