# Supplementary material for: Genetic structure of two sympatric gudgeon fishes (Xenophysogobio boulengeri and X. nudicorpa) in the upper reaches of Yangtze River Basin
Source: PeerJ. 2019 Aug 6;7:e7393. doi: 10.7717/peerj.7393 (PMC6688597; doi:10.7717/peerj.7393)
Supplement: Supplemental Information 8 — Genetic distance has been calculated by the formula: FST/(1-FST). [file peerj-07-7393-s008.docx]

|  | Cyt *b* | | | | | CR | | | | | SSR | | | | |
| --- | --- | --- | --- | --- | --- | --- | --- | --- | --- | --- | --- | --- | --- | --- | --- |
|  | JJ | YB | SF | YS | QW | JJ | YB | SF | YS | QW | JJ | YB | SF | YS | QW |
| JJ | 0 |  |  |  |  | 0 |  |  |  |  | 0 |  |  |  |  |
| YB | -0.005 | 0 |  |  |  | 0.064 | 0 |  |  |  | 0.006 | 0 |  |  |  |
| SF | 0.031 | 0.038 | 0 |  |  | 0.295 | 0.157 | 0 |  |  | -0.010 | -0.002 | 0 |  |  |
| YS | -0.003 | -0.006 | 0.072 | 0 |  | 0.013 | -0.007 | 0.163 | 0 |  | -0.001 | 0.002 | -0.013 | 0 |  |
| QW | 0.031 | 0.019 | 0.138 | -0.017 | 0 | 0.073 | -0.002 | 0.082 | -0.005 | 0 | -0.003 | 0.004 | -0.011 | 0.000 | 0 |
