# Supplementary material for: Genetic structure of two sympatric gudgeon fishes (Xenophysogobio boulengeri and X. nudicorpa) in the upper reaches of Yangtze River Basin
Source: PeerJ. 2019 Aug 6;7:e7393. doi: 10.7717/peerj.7393 (PMC6688597; doi:10.7717/peerj.7393)
Supplement: Supplemental Information 9 — Genetic distance has been calculated by the formula: FST/(1-FST). [file peerj-07-7393-s009.docx]

|  | Cyt *b* | | | | CR | | | | SSR | | | |
| --- | --- | --- | --- | --- | --- | --- | --- | --- | --- | --- | --- | --- |
|  | JJ | YB | QJ | PZH | JJ | YB | QJ | PZH | JJ | YB | QJ | PZH |
| JJ | 0 |  |  |  | 0 |  |  |  | 0 |  |  |  |
| YB | 0.124 | 0 |  |  | 0.289 | 0 |  |  | -0.028 | 0 |  |  |
| QJ | 0.555 | 0.05 | 0 |  | 0.667 | 0.072 | 0 |  | -0.001 | -0.038 | 0 |  |
| PZH | 1.222 | 1.667 | -0.158 | 0 | 1.488 | 1.252 | 0.006 | 0 | 0.075 | 0.042 | -0.029 | 0 |
