# Supplementary material for: Genetic structure of two sympatric gudgeon fishes (Xenophysogobio boulengeri and X. nudicorpa) in the upper reaches of Yangtze River Basin
Source: PeerJ. 2019 Aug 6;7:e7393. doi: 10.7717/peerj.7393 (PMC6688597; doi:10.7717/peerj.7393)
Supplement: Supplemental Information 10 [file peerj-07-7393-s010.docx]

|  |  | Groups | Prior maximal distance *P* |
| --- | --- | --- | --- |
| *X.boulengeri* | Cyt *b* | 1 | 0.001000 |
|  | CR | 43 | 0.001000 |
|  |  | 1 | 0.001292 |
| *X.nudicorpa* | Cyt *b* | 10 | 0.001000 |
|  |  | 6 | 0.001292 |
|  |  | 6 | 0.001668 |
|  |  | 3 | 0.002154 |
|  |  | 3 | 0.002783 |
|  |  | 2 | 0.003594 |
|  |  | 1 | 0.004642 |
|  | CR | 23 | 0.001000 |
|  |  | 4 | 0.001292 |
|  |  | 4 | 0.001668 |
|  |  | 4 | 0.002154 |
|  |  | 1 | 0.002783 |
| *X.boulengeri + X.nudicorpa* | Cyt *b* | 129 | 0.001000 |
|  |  | 7 | 0.001668 |
|  |  | 4 | 0.002783 |
|  |  | 2 | 0.004642 |
|  |  | 2 | 0.007743 |
|  |  | 2 | 0.012915 |
|  |  | 2 | 0.021544 |
|  |  | 2 | 0.035938 |
|  |  | 2 | 0.059948 |
|  |  | 2 | 0.100000 |
|  | CR | 66 | 0.001000 |
|  |  | 5 | 0.001668 |
|  |  | 2 | 0.002783 |
|  |  | 2 | 0.004642 |
|  |  | 2 | 0.007743 |
|  |  | 2 | 0.012915 |
|  |  | 2 | 0.021544 |
|  |  | 2 | 0.035938 |
|  |  | 2 | 0.059948 |
|  |  | 2 | 0.100000 |
