# Supplementary material for: Genetic structure of two sympatric gudgeon fishes (Xenophysogobio boulengeri and X. nudicorpa) in the upper reaches of Yangtze River Basin
Source: PeerJ. 2019 Aug 6;7:e7393. doi: 10.7717/peerj.7393 (PMC6688597; doi:10.7717/peerj.7393)
Supplement: Supplemental Information 11 [file peerj-07-7393-s011.docx]

|  | **JJ** | **YB** | **SF** | **YS** | | **QW** | **QJ** | **PZH** |
| --- | --- | --- | --- | --- | --- | --- | --- | --- |
| **JJ** |  |  |  |  |  | |  |  |
| Cyt *b* |  | 4.045 |  |  |  | | 0.901 | 0.409 |
| CR |  | 1.549 |  |  |  | | 0.750 | 0.336 |
| SSR |  | -17.741 |  |  |  | | -500.500 | 6.643 |
| **YB** |  |  |  |  |  | |  |  |
| Cyt *b* | -100.500 |  |  |  |  | | 9.917 | 0.300 |
| CR | 7.833 |  |  |  |  | | 6.963 | 0.399 |
| SSR | 82.833 |  |  |  |  | | -13.000 | 12.000 |
| **SF** |  |  |  |  |  | |  |  |
| Cyt *b* | 16.167 | 13.014 |  |  |  | |  |  |
| CR | 1.693 | 3.176 |  |  |  | |  |  |
| SSR | -50.500 | -250.500 |  |  |  | |  |  |
| **YS** |  |  |  |  |  | |  |  |
| Cyt *b* | -167.167 | -83.833 | 6.963 |  |  | |  |  |
| CR | 37.962 | -71.929 | 3.071 |  |  | |  |  |
| SSR | -500.500 | 249.500 | -38.962 |  |  | |  |  |
| **QW** |  |  |  |  |  | |  |  |
| Cyt *b* | 16.167 | 25.816 | 3.632 | -29.912 |  | |  |  |
| CR | 6.853 | -250.500 | 6.079 | -100.500 |  | |  |  |
| SSR | -167.167 | 124.500 | -45.955 |  |  | |  |  |
| **QJ** |  |  |  |  |  | |  |  |
| Cyt *b* |  |  |  |  |  | |  | -3.160 |
| CR |  |  |  |  |  | |  | 82.833 |
| SSR |  |  |  |  |  | |  | -17.167 |
| **PZH** |  |  |  |  |  | |  |  |
| Cyt *b* |  |  |  |  |  | |  |  |
| CR |  |  |  |  |  | |  |  |
| SSR |  |  |  |  |  | |  |  |
