# Supplementary material for: Genetic structure of two sympatric gudgeon fishes (Xenophysogobio boulengeri and X. nudicorpa) in the upper reaches of Yangtze River Basin
Source: PeerJ. 2019 Aug 6;7:e7393. doi: 10.7717/peerj.7393 (PMC6688597; doi:10.7717/peerj.7393)
Supplement: Supplemental Information 13 — Yes indicates the presence of an allele, No indicates the absence of an allele,–indicates insuffient sample. [file peerj-07-7393-s013.docx]

|  | **LT-C5** | **LT-C6** | **LT-C7** | **LT-D1** | **LT-D2** | **LT-D3** | **LT-D7** | **LT-D8** | **LT-D9** |
| --- | --- | --- | --- | --- | --- | --- | --- | --- | --- |
| JJ | Yes | No | No | Yes | No | No | No | Yes | No |
| YB | No | No | No | No | No | No | No | No | No |
| QJ | - | - | - | - | - | - | - | - | - |
| PZH | No | No | No | No | No | No | No | No | No |
